# ASP-2/*Trans*-sialidase chimeric protein induces robust protective immunity in experimental models of Chagas’ disease

**DOI:** 10.1038/s41541-023-00676-0

**Published:** 2023-05-31

**Authors:** Julia T. Castro, Rory Brito, Natalia S. Hojo-Souza, Bárbara Azevedo, Natalia Salazar, Camila P. Ferreira, Caroline Junqueira, Ana Paula Fernandes, Ronnie Vasconcellos, Jamille M. Cardoso, Rodrigo D. O. Aguiar-Soares, Paula M. A. Vieira, Cláudia M. Carneiro, Bruno Valiate, Cristiane Toledo, Andres M. Salazar, Otávia Caballero, Joseli Lannes-Vieira, Santuza R. Teixeira, Alexandre B. Reis, Ricardo T. Gazzinelli

**Affiliations:** 1 Centro de Tecnologia em Vacinas, Universidade Federal de Minas Gerais, Parque Tecnológico de Belo Horizonte, Belo Horizonte, Brazil; 2 Centro de Pesquisas Rene Rachou, Fundação Osvaldo Cruz, Rio de Janeiro, Brazil; 3 Plataforma de Medicina Translacional, Fundação Oswaldo Cruz-Faculdade de Medicina de Ribeirão Preto, Universidade de São Paulo, São Paulo, Brazil; 4 Universidade Federal de Ouro Preto, Ouro Preto, Brazil; 5 Universidade Federal de São Paulo, São Paulo, Brazil; 6 Oncovir, Inc, Washington, USA; 7 Orygen, LTDA, Sao Paulo, Brazil; 8 Fundação Osvaldo Cruz, Rio de Janeiro, Brazil

**Keywords:** Protein vaccines, Parasitic infection

## Abstract

Immunization with the Amastigote Surface Protein-2 (ASP-2) and *Trans*-sialidase (TS) antigens either in the form of recombinant protein, encoded in plasmids or human adenovirus 5 (hAd5) confers robust protection against various lineages of *Trypanosoma cruzi*. Herein we generated a chimeric protein containing the most immunogenic regions for T and B cells from TS and ASP-2 (TRASP) and evaluated its immunogenicity in comparison with our standard protocol of heterologous prime-boost using plasmids and hAd5. Mice immunized with TRASP protein associated to Poly-ICLC (Hiltonol) were highly resistant to challenge with *T. cruzi*, showing a large decrease in tissue parasitism, parasitemia and no lethality. This protection lasted for at least 3 months after the last boost of immunization, being equivalent to the protection induced by DNA/hAd5 protocol. TRASP induced high levels of *T. cruzi-*specific antibodies and IFNγ-producing T cells and protection was primarily mediated by CD8^+^ T cells and IFN-γ. We also evaluated the toxicity, immunogenicity, and efficacy of TRASP and DNA/hAd5 formulations in dogs. Mild collateral effects were detected at the site of vaccine inoculation. While the chimeric protein associated with Poly-ICLC induced high levels of antibodies and CD4^+^ T cell responses, the DNA/hAd5 induced no antibodies, but a strong CD8^+^ T cell response. Immunization with either vaccine protected dogs against challenge with *T. cruzi*. Despite the similar efficacy, we conclude that moving ahead with TRASP together with Hiltonol is advantageous over the DNA/hAd5 vaccine due to pre-existing immunity to the adenovirus vector, as well as the cost-benefit for development and large-scale production.

## Introduction

Chagas’ disease (CD) is caused by a long-lived infection with *Trypanosoma cruzi* and affects approximately six to eight million chronically infected individuals in Latin America^1,2^. Although natural transmission is controlled in many countries of Latin America, outbreaks secondary to contamination of food or beverages by triatomine (kissing bug) feces containing infective *T. cruzi* metacyclic trypomastigotes have often been reported^3^. While natural transmission to humans is restricted to Latin America, due to immigration, the US today has approximately 300,000 patients with CD^4^. In addition, the autochthonous CD has been reported in southeastern Texas^5^. These alarming numbers combined with the severity of the disease and risk of parasite transmission by blood transfusion and organ transplantation, also makes CD a highly relevant public health problem in this and other countries outside of Latin America^1,2^. Unfortunately, this disease is largely neglected by public health authorities and the pharmaceutical industry^6,7^. This scenario is worsened by the lack of a prophylactic vaccine; besides, while available effectives drugs, the treatment can cause adverse effects and some *T. cruzi* strains are naturally resistant to chemotherapy for CD^8,9^.

Our research group has focused on developing a preventive and therapeutic vaccine against infection with *T. cruzi*. In these studies, we used the members of the *trans*-sialidase family amastigote surface protein-2 (ASP-2) and *trans*-sialidase (TS) as vaccine candidates. We have used adjuvanted proteins, plasmids, influenza PR8 vector, as well as the human Adenovirus 5 (hAd5) encoding either ASP-2 or TS proteins (hAd-ASP-2 and hAd-TS, respectively)^10–27^. The most effective protocol used in our lab employs priming with a combination of plasmids encoding ASP-2 and TS followed by a boost with the hAd-ASP-2 and hAd-TS, 21 days apart. While the vaccination with each of the single recombinant proteins was not as effective, the use of a vaccination protocol that employs two different plasmid DNAs followed by two distinct hAd5 is not cost-effective. Therefore, we thought of developing a vaccine that uses a fusion protein containing both ASP-2 and TS.

In this study, we first engineered a chimeric protein, called TRASP, that contains the segments of TS and ASP-2 with most of the potential CD8^+^ T cell epitopes and the C-terminal repeats of the TS (SAPA), which is a potent B cell epitope. Then we tested TRASP associated to the Toll-Like Receptor 3 and MDA5 ligand, namely polyinosinic-polycytidylic (Poly-IC) stabilized with poly-lysine and carboxymethylcellulose (Poly-ICLC). Also named Hiltonol, Poly-ICLC is an adjuvant that has been used in multiple clinical trials as an immune-stimulatory agent to treat cancer^28–41^. Our results show that this formulation is highly efficient for inducing both TRASP-specific antibodies and IFN-γ production by CD4^+^ T and CD8^+^ T cells. Importantly, it provides strong protection against *T. cruzi* challenge in mice, up to 90 days post-immunization. Finally, this formulation is highly immunogenic, presents limited side effects and protects dogs against experimental challenges with *T. cruzi*. Thus, we consider that this vaccine prototype should be moved toward the development of a vaccine for CD.

## Results

### Engineering the chimeric recombinant protein named TRASP

**Fig. 1:**
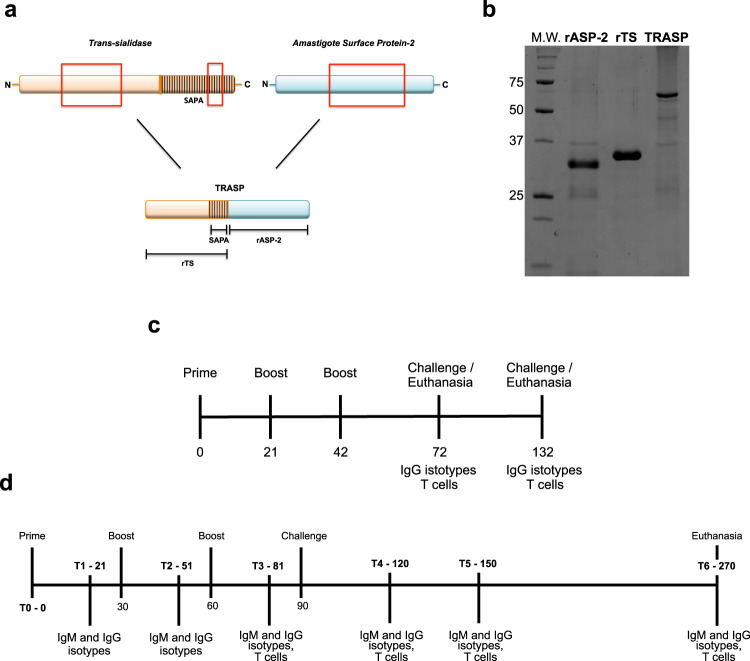
Construction of the chimeric protein TRASP and immunization schedule. **a** The immunogenic regions of TS, ASP-2 and three SAPA-repeats were joined into a single sequence, giving rise to a chimeric protein named TRASP. **b** TRASP and the isolated fragments rTS and rASP-2 were expressed in *E. coli* and purified. Proteins’ purification was confirmed by SDS-PAGE. After that, the potential of TRASP as a vaccine was tested in murine (**c**) and canine (**d**) models.

The amino acid sequences of TS and ASP-2 proteins were defined by screening human leukocyte antigen (HLA)-ABC-binding epitopes using the programs SYFPEITHI, Bimas, and IEDB. The epitopes that received the best scores for each HLA allele were selected. In order to find immunodominant epitopes, the frequency in which the same peptide appeared in the top score for different HLA-ABC alleles was analyzed. Because *T. cruzi* has a high genetic diversity between strains, especially in the TS multigene family^42^, we also looked for conserved immunogenic epitopes among different discrete typing units (DTUs) (Supplementary Tables 1, 2). Using these strategies, it was possible to detect potentially immunogenic regions in both proteins. The selected segments of ASP-2 and TS also included dog’s MHC (DLA) (Supplementary Tables 3, 4) and mice H2-K^b^ and H2-K^d^-binding epitopes^18,23^. These regions from TS associated with three SAPA-repeats and ASP-2 were joined into a single sequence, giving rise to the chimeric protein named TRASP (Fig. 1a). For comparison purposes, the selected regions from TS and ASP-2 (named rTS and rASP-2) were used to evaluate antibody and T cell responses. The relative molecular mass predicted for the recombinant proteins were TRASP (≅ 62), rTS (≅ 32), and rASP-2 (≅ 30) (Fig. 1b and Supplementary Fig. 1).

### Immunization protocols

In order to investigate the potential of TRASP as a vaccine, C57BL/6 mice and dogs were immunized with this protein supplemented with Hiltonol® [Poly-ICLC] (TRASP-pICLC). CpG associated with Alum was used as our control adjuvant in the mouse experiments. The experimental schedule is illustrated in Fig. 1c. First, the animals were administered with three doses 21 days apart. Thirty or ninety days after the last immunization, a group of mice was euthanized and another group was challenged with *T. cruzi* Y strain. In some experiments, we included a group of mice receiving a prime dose with plasmids (DNA TS/ASP) and a boost of Ad5 containing TS and ASP-2 sequences (Ad TS/ASP), 30 days apart.

We also evaluated this potential vaccine in the canine model, which is considered the best model to study Chagas’ disease by the World Health Organization (1984). Infected dogs develop a cardiac disease very similar to humans in both acute and chronic stages^43^, and also present a similar profile of IgM and IgG production^44^. As in humans, CD8^+^ T cells and IFN-γ are increased in infected dogs^45^, whereas an unbalanced proinflammatory response correlates with cardiomyopathy severity^46^. The animals were immunized with three doses of TRASP-pICLC or with a prime of DNA TS/ASP and one boost of Ad TS/ASP, as shown in Fig. 1d. Dogs had their blood collected between the doses (T1–T3) and they were challenged with *T. cruzi* Berenice-78 strain thirty days after the last administration. There were also blood-collection points after the challenge (T4–T6). Finally, the animals were euthanized 180 days after the challenge.

### Immunization with TRASP adjuvanted with Poly-ICLC elicits a robust immune response and protects mice against *T. cruzi* infection

Thirty days after the last boost, splenocytes were collected and stimulated with rTS, rASP-2, or TRASP. As expected, splenocytes derived from mice immunized with TRASP secreted high levels of IFN-γ, in comparison to the control groups (received adjuvant only and non-immunized) (Supplementary Fig. 2a). It is important to highlight that rTS and rASP-2 stimulated the IFN-γ production by splenocytes with the same intensity. On the other hand, IL-10 production was low in all immunized groups under specific stimuli (Supplementary Fig. 2b).

When compared with mice that received TRASP + CpG + Alum, splenocytes from animals immunized with TRASP-pICLC produced similar levels of IFN-γ after stimulation with rASP-2, rTS, or TRASP. Moreover, there was no statistically significant difference between the secretion of IL-10 in both groups (Supplementary Fig. 2b). The same was observed in the humoral response, and Poly-ICLC showed to be as good as CpG + Alum to induce the production of TS- and ASP-2-specific IgGs, when administered with TRASP (Supplementary Fig. 2c).

Regarding the ability to protect against *T. cruzi* infection, it was observed that TRASP adjuvanted with Poly-ICLC was as efficient as TRASP + CpG + Alum, as demonstrated by the lower parasite load in the blood. On the seventh-day post-infection, there was a 96.7% reduction in parasitemia of TRASP-immunized mice compared to the non-immunized group (PBS group) (Supplementary Fig. 2d). Both groups immunized with TRASP showed a 100% survival rate. PBS control groups presented 66%, the adjuvant controls Poly-ICLC 80%, and CpG + Alum 83% of survival (Supplementary Fig. 2e). Because Poly-ICLC has shown to be a good adjuvant and has been successfully tested in various clinical trials for cancer therapy and HIV vaccines^28–41^, we decided to maintain it in our formulation for the next assays.

Because it is known that SAPA is a highly immunogenic region that can drive humoral response leading to decreased levels of antibodies against the catalytic domain of TS and immune evasion^47^ we measured the levels of IgG anti-SAPA and anti-TS without SAPA. As depicted in Supplementary Fig. 2f, g, mice immunized with TRASP adjuvanted with Poly ICLC produce high levels of IgG antibodies against both regions; showing that the SAPA-repeats included in TRASP are not preventing the humoral response against TS catalytic domain. Although the main targets of lytic antibodies from humans are related to *T. cruzi* mucins^48^, we investigated whether immunization with TRASP-pICLC could induce lytic antibodies in mice. In contrast to antibodies from naïve mice that were infected with *T. cruzi* at 15 days post-infection (DPI), antibodies present in the sera of TRASP-immunized animals did not show trypanolytic activity (Supplementary Fig. 2h).

### Immunization with TRASP-pICLC confers protection against different *T. cruzi* strains

Due to the extensive genetic diversity among *T. cruzi* strains, it is important that a vaccine against CD can elicit cross-protection against different isolates. To assess this issue, we challenged TRASP-immunized mice with CL-Brener (TcVI) or Be-78 (TcII) strains. As shown in Supplementary Fig. 3, immunization with TRASP-pICLC provided protection against both strains, demonstrated by significantly lower parasitemia in immunized mice in comparison with the control group. This data supports our in silico findings that the TRASP sequence has highly conserved immunogenic epitopes among different DTUs (Supplementary Tables 1, 2).

### TRASP associated with Poly-ICLC provides protection in mice after 90 days of immunization

**Fig. 2:**
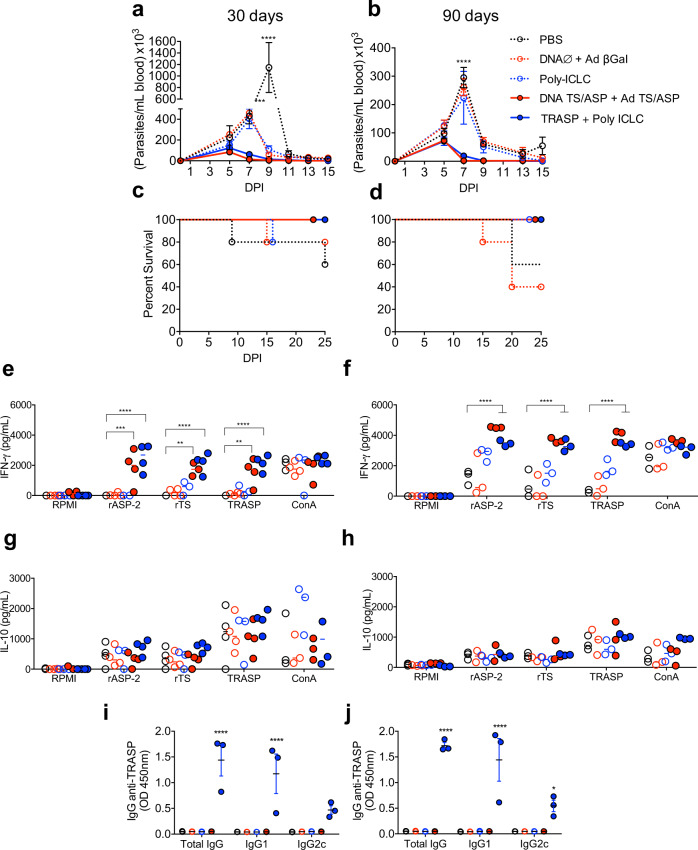
Analysis of prolonged protection. **a**, **b** Immunized mice were challenged with 10^4^ blood trypomastigotes of *T. cruzi* Y strain at 30 (**a**) or 90 (**b**) days after the last vaccine dose, and parasitemia was followed by 15 days post-infection (DPI), mean ± SEM. Mice survival was evaluated until 25 DPI in both time points (**c**, **d**). **e**–**h** The levels of IFN-γ (**e**, **f**) and IL-10 (**g**, **h**) produced by splenocytes were evaluated after culture with specific stimuli at 30 (**e**, **g**) and 90 (**f**, **h**) days post-immunization. Additionally, anti-TRASP IgG isotypes were evaluated in mice sera at 30 (**i**) and 90 (**j**) days post-immunization. The statistical analysis of parasitemia and cytokine levels was carried out using two-way ANOVA and Tukey’s multiple comparisons test. The statistical analysis of survival was performed using the log-rank test. Data were representative of two independent experiments (*n* = 4–6 mice per group). *p < 0.05; ***p < 0.001; ****p < 0.0001.

In order to evaluate whether this immunization protocol affords prolonged protection, mice were immunized according to the same schedule (Fig. 1c), but the groups were challenged at two different time points: 30 or 90 days after the last boost. For comparison, a group immunized with our gold standard vaccination protocol DNA TS/ASP + Ad TS/ASP was included in this experiment. As observed in Fig. 2, mice immunized with our formulation TRASP-pICLC achieved similar levels of protection to the mice that received the genetic vaccination, as shown by the lower parasitemia compared with control groups (Fig. 2a, b). Importantly, both immunizations were efficient even when the challenge was performed at 90 days post-vaccine administration (Fig. 2b). Besides that, 100% of immunized animals have survived at both time points (Fig. 2c, d). These data suggest that TRASP-pICLC is able to induce prolonged protection.

Next, mice splenocytes were collected at 30 or 90 days after the last boost and were analyzed for IFN-γ / IL-10 production, and antibody isotypes in the sera were also evaluated (Fig. 2e–j). The results presented in Fig. 2e, f demonstrate that splenocytes from immunized mice are able to produce high levels of IFN-γ under specific stimuli (rTS, rASP-2, or TRASP) at 30 days as well as after 90 days of vaccination, and there was no difference between either immunization protocols. The production of IL-10 (Fig. 2g, h) was low and similar in all groups, vaccinated or not. We also evaluated antigen-specific antibodies in mice sera, and surprisingly, only TRASP-immunized mice showed high levels of total IgG, IgG1, and IgG2c, while animals that received DNA TS/ASP + Ad TS/ASP showed no antibody production in each time point (Fig. 2i, j).

**Fig. 3:**
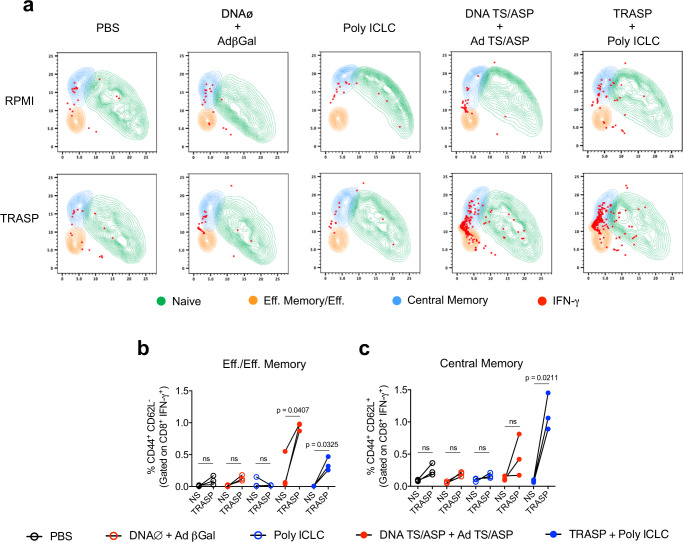
Immunophenotyping of CD8^+^ T lymphocytes in immunized mice. After 90 days of immunization, mice splenocytes were stimulated with TRASP for 18 h or left unstimulated (RPMI only) and were then characterized by flow cytometry. **a** UMAP projection of IFN-γ production by CD8^+^ T lymphocytes compartments, separated in Naïve (CD8^+^ CD44^−^ CD62L^−^), effector/effector memory (CD8^+^ CD44^+^ CD62L^−^), and central memory (CD8^+^ CD44^+^ CD62L^+^). **b**, **c** The frequency of IFN-γ-producing CD8+ effector/effector memory (**b**) or central memory T cells (**c**). The statistical analysis was carried out using paired *t*-test. Data were representative of two independent experiments (*n* = 3 mice per group). ns non-significant.

To understand which cells are being activated by each immunization protocol, we analyzed the immune response by flow cytometry of TRASP-stimulated splenocytes after 90 days of vaccination. A Uniform Manifold Approximation and Projection (UMAP) was generated to analyze IFN-γ production by CD8^+^ T cells (Supplementary Fig. 4). As shown in Fig. 3a–c, mice administered with TRASP-pICLC showed an increase in IFN-γ^+^ CD8^+^ T effector/effector memory and central memory cells after stimulation with TRASP. The DNA TS/ASP + Ad TS/ASP group presented a robust expansion of IFN-γ−producing effector/effector memory CD8^+^ T cells only.

**Fig. 4:**
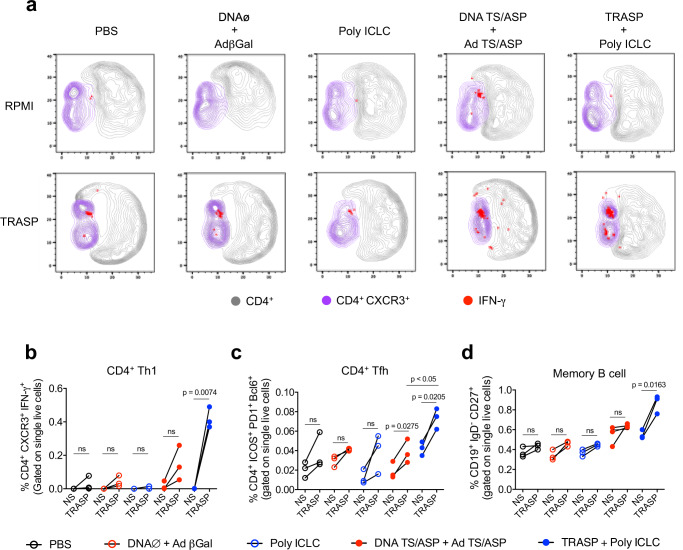
Characterization of CD4^+^ T and B lymphocytes elicited by vaccination. In vitro-stimulated splenocytes were immunophenotyped by flow cytometry after 90 days of immunization. **a** UMAP projection showing the production of IFN-γ by CD4^+^ Th1 cells (CD4^+^ CXCR3^+^). **b** Frequency of CD4^+^ CXCR3^+^ IFN-γ^+^ **c** CD4^+^ Tfh (CD4^+^ ICOS^+^ PD-1^+^ Bcl6^+^), and **d** class-switched memory B cells (CD19^+^ IgD^−^ CD27^+^). The statistical analysis was carried out using paired *t*-test. Data were representative of two independent experiments (*n* = 3 mice per group). ns non-significant.

We also looked at the frequency of Th1 cells (CD4^+^ CXCR3^+^ IFN-γ^+^) in response to in vitro stimulation with TRASP (Supplementary Fig. 5a and Fig. 4a, b), and it was detected a raise of this population in TRASP-immunized mice, but not in animals administered with DNA TS/ASP + Ad TS/ASP. The same was observed for Tfh (CD4^+^ ICOS^+^ PD-1^+^ Bcl6^+^) and class-switched memory B cells (CD19^+^ IgD^−^ CD27^+^), which were induced to proliferate after incubation with TRASP only in mice that received TRASP-pICLC (Supplementary Fig. 5b, c and Fig. 4c, d).

### Role of CD8^+^ T cells as well as IFN-γ on TRASP + Poly ICLC-induced protective immunity

**Fig. 5:**
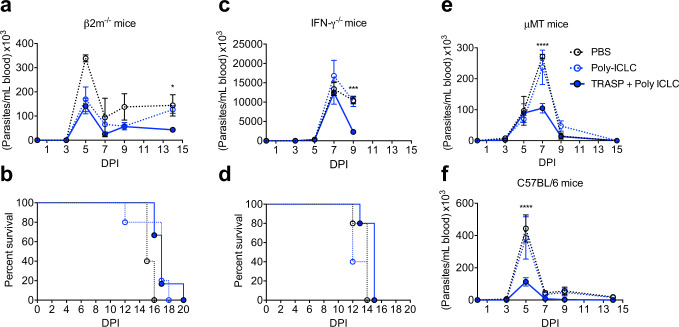
Importance of T and B lymphocytes in the protection of immunized mice against *T. cruzi* infection. β-2m^−/−^ (**a**, **b**), IFN-γ^−/−^ (**c**, **d**), μMT (**e**), and C57BL/6 (**f**) mice were immunized with TRASP-pICLC and challenged with *T. cruzi*. Parasitemia (**a**, **c**, **e**, **f**) and survival (**b**, **d**) were measured for 9 to 20 days, depending on the model. Statistics of parasitemia was calculated by using two-way ANOVA and Tukey’s multiple comparisons test. Data were representative of two independent experiments, mean ± SEM (*n* = 4–6 mice per group). *p < 0.05; ***p < 0.001; ****p < 0.0001.

Given these results, we decided to evaluate if the protection elicited by TRASP-pICLC is dependent on CD8^+^ T cells. To assess this, we immunized β-2m^−/−^ mice, which lacks CD8^+^ T cells, and challenged them with *T. cruzi*. The animals were not protected against the infection, despite they presented a lower parasitemia than the PBS group at 5 DPI, there was no difference when compared to the group that received Poly-ICLC only. After 14 days of challenge, we observed a significant decrease in the parasite load of TRASP-immunized mice, but all β-2m^−/−^ mice succumbed to infection within 20 DPI (Fig. 5a, b).

To confirm that the vaccination with TRASP-pICLC elicits, especially a Th1 immune response, dependent on IFN-γ secretion, IFN-γ^−/−^ mice were immunized and challenged with *T. cruzi*. As expected, all animals showed extremely high levels of parasitemia and were dead at 15 DPI, regardless they were immunized or not (Fig. 5c, d). Therefore, it is suggested that the immune response induced by TRASP-pICLC is highly dependent on IFN-γ. On the other hand, immunized B cell-deficient mice (μMT) with TRASP-pICLC showed a prolonged parasitemia when compared to the vaccinated wild type, but surprisingly, circulating parasites were controlled by 9 DPI (Fig. 5e), in comparison with 7 DPI in C57BL/6 mice (Fig. 4f). No mortality was observed in μMT mice challenged with *T. cruzi*. These data indicate that despite eliciting a great production of antibodies, the protective response induced by TRASP-pICLC is primarily dependent on T cell-mediated immunity.

In conclusion, our data indicate that immunization with TRASP-pICLC induces IFN-γ production by CD8^+^ T and CD4^+^ T cells. Although immunization also stimulates the proliferation of Tfh and memory B cells, protective immunity seems to be mostly dependent on CD8^+^ T cells and IFN-γ. While immunization with DNA TS/ASP + Ad TS/ASP induced a stronger response of effector/effector memory CD8^+^ T cells compared to mice immunized with TRASP, the level of protection was similar between mice immunized with TRASP + Poly ICLC versus genetic vaccination.

### Immunization with TRASP associated with Poly-ICLC has low toxicity and side effects in dogs

Because we obtained promising results in the murine model, we decided to test our formulation in dogs, which are considered an appropriate model to study human Chagas’ disease [74,75]. The animals were immunized according to the schedule presented in Fig. 1d. First, we analyzed whether the vaccine administration would cause toxicity, and therefore several parameters were evaluated 24–72 h after each dose, such as weight loss, fever, edema, local pain, rash, and skin peeling (Supplementary Table 5). No local or systemic alterations on the animals were detected after the first dose, in any of the groups. However, after the second dose, it was noticed the emergence of edema in animals that received Poly-ICLC (1/6), DNA TS/ASP + Ad TS/ASP (4/9), and TRASP-pICLC (6/9). No other symptoms were observed. After the third dose, there was an appearance of local edema in dogs from those same groups, and also local pain in one animal administered with DNA TS/ASP + Ad TS/ASP and two with TRASP-pICLC (2/9). Nevertheless, all of these were mild alterations disappeared after 24 h. According to these results, both immunizations present low toxicity and, consequently are safe.

### Vaccination with TRASP associated with Poly-ICLC is highly immunogenic in dogs

**Fig. 6:**
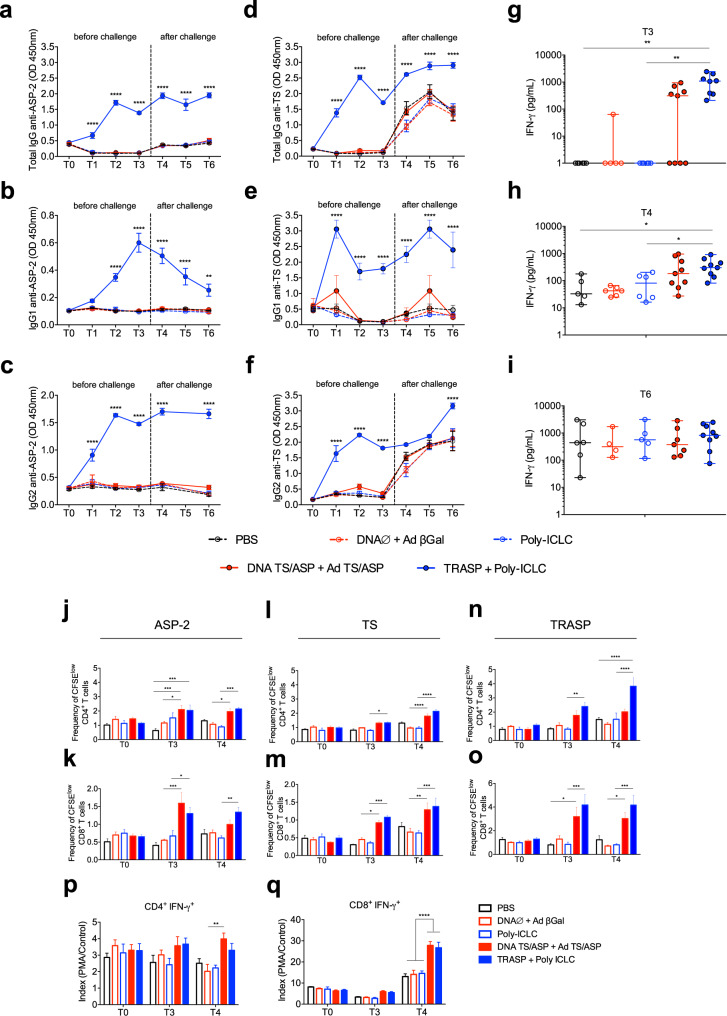
Humoral and cellular immune response induced by vaccination in dogs. Elevated levels of IgG isotypes anti-ASP-2 (**a**–**c**) and anti-TS (**d**–**f**) were detected in the sera from dogs immunized with TRASP-pICLC in different time points. **g**–**i** IFN-γ production was analyzed in PBMC’s culture supernatant stimulated with TRASP at T3 (**g**), T4 (**h**), and T6 (**i**). **j**–**o** The proliferation of CD4^+^ and CD8^+^ T cells under specific stimuli of ASP-2 (**j**, **k**), TS (**l**, **m**), and TRASP (**n**, **o**) was evaluated at T0, T3, and T4. Intracellular staining of IFN-γ on CD4^+^ (**p**) and CD8^+^ (**q**) T lymphocytes was assessed by flow cytometry and is represented by index (PMA/Control). All the statistical analysis was carried out using two-way ANOVA and Tukey’s multiple comparisons test, except data from IFN-γ detection, which was analyzed by Kruskal–Wallis and Dunn’s multiple comparisons test. Mean ± SEM. *n* = 6–9 dogs per group. *p < 0.05; **p < 0.01; ***p < 0.001; ****p < 0.0001.

To evaluate the humoral immune response induced by vaccination, IgM and IgG antibodies were measured in dogs’ sera. On early time points (T1–T3), we identified an increase of specific IgM anti-ASP-2, anti-TS, and anti-TRASP in animals immunized with TRASP-pICLC, compared with the genetic vaccination and control groups (Supplementary Fig. 6). Total IgG and IgG1/2 isotypes anti-ASP-2 and anti-TS were detected at remarkable levels in the animals vaccinated with TRASP associated with Poly-ICLC (Fig. 6a–f). Importantly, high rates of IgG isotypes persisted in these animals after the challenge. Surprisingly, before the challenge, dogs immunized with DNA TS/ASP + Ad TS/ASP did not induce detectable levels of antigen-specific IgGs. After the challenge, we noticed that, similar to the non-vaccinated groups, it was detected high levels of IgGs anti-TS, but not anti-ASP-2, suggesting that the TS-specific antibodies were induced by *T. cruzi* infection.

Regarding the cellular immune response, it was observed that PBMCs from all dogs immunized with TRASP-pICLC were strongly induced to secrete IFN-γ under stimulation with TRASP after 21 days of vaccination. In contrast, not all dogs immunized with DNA TS/ASP + Ad TS/ASP have PBMCs that produced this cytokine (Fig. 6g). Thirty days post-challenge with *T. cruzi* (T4), IFN-γ production by PBMCs stimulated with TRASP was elevated in all dogs. Still, the levels were higher in TRASP-immunized animals compared to genetically immunized dogs (Fig. 6h). In contrast, after 180 days of infection (T6), all animals responded to TRASP stimulus, regardless of the immunization (Fig. 6i).

We also assessed the ability of T lymphocytes to proliferate after stimulation with the recall antigens ASP-2, TS, or TRASP. As depicted in Fig. 6j, k, ASP-2 induced the expansion of CD4^+^ and CD8^+^ T cells from immunized dogs at 30 days post-vaccination (T3). Thirty days after the challenge (T4), CD4^+^ T cells maintained the ability to proliferate under ASP-2 stimulus in both immunized groups, while the proliferation of CD8^+^ T cells persisted only in TRASP-immunized animals. Stimulation with TS elicited a slight expansion of CD4^+^ T cells from dogs vaccinated with TRASP-pICLC at T3 (Fig. 6l). On the other hand, it was detected a significant increase of CD8^+^ T cells derived from both immunized groups, compared to controls (Fig. 6m). Thirty days post-challenge, the proliferation was observed on CD4^+^ and CD8^+^ populations from animals vaccinated with TRASP-pICLC and DNA TS/ASP + Ad TS/ASP (Fig. 6l, m). In turn, TRASP stimulation has led to a significant expansion of CD4^+^ T lymphocytes, especially at T4, but only in TRASP-immunized dogs (Fig. 6n). Otherwise, TRASP-stimulated CD8^+^ T cells were augmented in both vaccinated groups at T3 and T4 (Fig. 6o). Interestingly, TRASP stimulus elicited a greater proliferation of CD8^+^ T lymphocytes than ASP-2 and TS. In dogs administered with TRASP-pICLC, the frequency of these cells were approximately four times higher under TRASP stimulation than the other antigens. Additionally, we assessed the production of IFN-γ on CD4^+^ and CD8^+^ T lymphocytes by flow cytometry (Fig. 6p, q). Substantial differences were observed after 30 days of infection, when there was an elevation of CD4^+^ IFN-γ^+^ T cells in the animals vaccinated with DNA TS/ASP + Ad TS/ASP, and a robust increase of CD8^+^ IFN-γ^+^ T cells in both immunized groups. According to this data, it is suggested that the major source of vaccine-induced IFN-γ is CD8^+^ T lymphocytes.

### Immunization with TRASP adjuvanted with Poly-ICLC protects dogs against *T. cruzi* infection

**Fig. 7:**
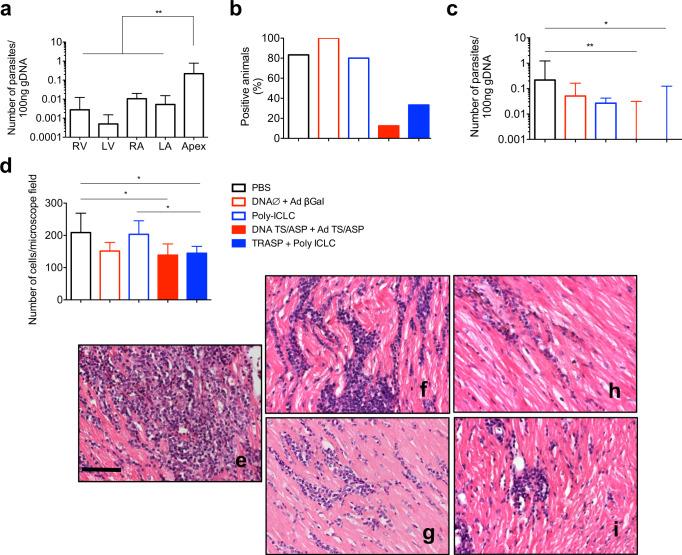
Evaluation of protection against *T. cruzi* infection. The parasite load in the heart was measured by qPCR (**a**) and the apex section, the most parasite-abundant, was selected to investigate the difference between the groups (**b**, **c**). It was analyzed the percentage of positive animals (**b**) and the number of parasites/100 ng of gDNA (**c**). Histopathological analysis showed an increase in inflammatory process in non-vaccinated dogs, represented by the number of cells per microscope field (**d**) and photomicrographs of the groups PBS (**e**), Poly-ICLC (**f**), DNA∅ + AdβGal (**g**), TRASP-pICLC (**h**), and DNA TS/ASP + Ad TS/ASP (**i**). **a**, **d** Mean ± SEM, **c** median ± range. The statistical analysis of the parasite load was assessed by Mann–Whitney test and histopathology was analyzed by one-way ANOVA and Tukey’s multiple test. *n* = 6–9 dogs per group. *p < 0.05; **p < 0.01.

Finally, we evaluated the antiparasitic response elicited by the vaccines. Dogs were euthanized 180 days after the challenge and had their heart collected for quantification of parasite load by qPCR. The heart was sectioned in the right and left atrium, right and left ventricle, and apex. Among these sections, the apex was the most parasite-abundant, while in the other heart areas *T. cruzi* DNA was detected in low levels (Fig. 7a). Therefore, the apex was used to compare the parasite load between the groups. As shown in Fig. 7b, only 12.5 and 33.3% of DNA TS/ASP + Ad TS/ASP and TRASP-pICLC-immunized dogs, respectively, presented detectable parasite DNA. On the other hand, control groups showed at least 80% of positive animals. Also, the number of parasites/100 ng of gDNA was significantly lower in both vaccinated groups, as depicted in Fig. 7c. Besides, the histopathology showed an intense inflammatory process in the left ventricle of unvaccinated animals, represented by an increase in the number of cells/microscope field (Fig. 7d–g). Otherwise, dogs immunized with DNA TS/ASP + Ad TS/ASP or TRASP-pICLC exhibited a discrete and focal inflammatory process (Fig. 7h, i). These results indicate that immunization with TRASP-pICLC is not only immunogenic, but also protective against *T. cruzi* infection.

## Discussion

Over the last 20 years, we focused on two antigens as vaccine candidates for CD^10–27^. These antigens are an amastigote surface protein, ASP-2, and the virulence factor named TS, which is encoded by a large gene family primarily expressed by the trypomastigotes^49,50^. This strategy was designed to aim for a vaccine that targets both the intracellular amastigote and the extracellular trypomastigote stages by inducing CD8^+^ cytotoxic T cells as well as antibodies, respectively^51^. Indeed, we found that the vaccines that contained both antigens were more effective in mice. These recombinant antigens associated with different adjuvants, encoded in plasmids or in the non-replicative adenovirushAd5 induced strong protective immunity^10–27^. While most of these formulations induced protection, we established that a heterologous prime-boost protocol using a mixture of plasmid DNA encoding ASP-2 and TS followed by a combination of hAd-ASP-2 and hAd-TS was the most effective protocol for inducing protective immunity. This vaccine formulation was highly efficacious both in preventive^19,23,26^ and therapeutic protocols^20^. In addition, it was protective in mice with different genetic backgrounds^12,19,23^ or challenged with different lineages of *T. cruzi*^11,23^. Here, we designed a TS/ASP-2 fusion protein (TRASP) based on human T cell epitopes and the highly immunogenic B cell epitope (SAPA) present in TS^49,50^. When associated with the adjuvant Poly-ICLC^28,29^ TRASP is highly immunogenic and provided strong protection in mice and dogs experimentally infected with *T. cruzi*.

After acute infection, individuals infected with *T. cruzi* develop a strong immune response and the disease evolves into a chronic asymptomatic stage. Thirty percent of these patients show barely detectable parasitemia, but develop a cardiomyopathy or the digestive form of disease^52^. In post-mortem studies, it was shown that tissue parasitism is also scarce in patients with chronic CD to the point that a *T. cruzi*-induced autoimmunity was evoked to explain the heart inflammatory processes in the absence of parasites^53^. However, by using more sensitive techniques, such as immunocytochemistry and PCR, a clear association between parasite and tissue inflammation has been shown, both in patients and mice experimentally infected with *T. cruzi*^54^. In addition, different studies suggest that severe chronic disease is positively associated with parasite load (parasitemia) during acute infection. Hence, the current consensus is that the inflammatory processes found in chronic patients is elicited by the presence of very few parasites and is not an autoimmune disease^54^. This is all to say that infection with *T. cruzi* induces a strong anti-parasite immune response that is highly effective in controlling the parasite load, but not enough to eradicate the infection in chronically infected hosts^51^. In the future, we intend to explore the efficacy of this vaccine in a therapeutic protocol to treat chronic Chagas’ disease.

Unfortunately, there is no available vaccine for CD and little funding is available to support the development of a vaccine. Nevertheless, several antigens or live attenuated and even CRISPR-edited parasites have been tested as vaccine candidates in experimental models^55^. Among the vaccine candidates different groups have used the TS, ASP-2, Tc24, cruzipain, members of the TcG family and other antigens^10–21^. For a complete list, please see reviews by Malchiodi^56^ and Garg^57^. As an attenuated vaccine, the CL-14 clone that is naturally deficient in expression of complete active TS and the CRISPR-edited cell line in which active TS genes have been disrupted, induces a strong immunity to challenge with the parental CL strain or the virulent clone CL-Brener or the Y strain^58–60^.

In a recent study, a fusion protein containing ASP-2, TS, and the cruzipain antigens was shown to induce a high degree of protection in mice. The authors used 3′5′-c-di-AMP as an adjuvant, an agonist cGAS, and showed that STING, a mediator of Type I IFN induced by cytosolic receptors for viral DNA and RNA, is necessary to induce protective immunity by this vaccine formulation^61^. Here, we focused in the ASP-2 and TS regions that have more abundant T cell epitopes and included a SAPA from TS antigen as a strong B cell epitope. In addition, we used an improved version of Poly (IC), which is encapsulated to assure RNA stability, and also limits the toxicity of this adjuvant. Poly-ICLC activates TLR3 and MDA5, as well as other cytosolic receptors for viral RNA. It has been produced in GMP grade and is used in various clinical trials together with anti-cancer and anti-viral vaccines^28–41^.

We used the plasmid DNA and replication-deficient adenovirus expressing ASP-2 and TS prime-boost protocol as our gold standard vaccine. When adjuvanted with Poly-ICLC, TRASP induced a strong production of IFN-γ, but not IL-10, by CD4^+^ and CD8^+^ T cells as well as activation of Tfh cells and B lymphocytes to produce high levels of antigen-specific IgG2c antibodies. The humoral immune response induced by the chimeric protein adjuvanted with Poly-ICLC was very robust, in contrast with the plasmid/adenovirus protocol that was not effective in this matter.

It is well known that CD8^+^ T cells, by producing IFN-γ and killing infected cells, play a central role in resistance to *T. cruzi*^14,22,62,63^. As expected, IFN-γ KO mice vaccinated with either protocol were highly susceptible to *T. cruzi*. Although the formulation using the replication-deficient adenovirus expressing ASP-2 and TS were more efficient in inducing IFN-γ^+^ effector/effector memory CD8^+^ T cells, mice immunized with TRASP-pICLC also showed an expansion of these cells, along with IFN-γ^+^ central memory CD8^+^ T cells. Consistently, the protection induced by the TRASP-pICLC formulation was highly dependent on IFN-γ, but also dependent on CD8^+^ T cells and less dependent on B lymphocytes and antibody production.

Finally, we report that immunization with TRASP-pICLC is highly immunogenic, nontoxic, and induced protection in dogs. It induced higher levels of anti-TS and anti-ASP-2 antibodies, a higher CD4^+^ Th1, and in contrast to mice, a slightly higher CD8^+^ T cell response, when compared to the plasmid/adenovirus vaccine. The immunization with TRASP-pICLC also induces a more robust immunity in dogs that may last longer, since, different from mice immunized with plasmid/adenovirus, both T and B cell compartments are engaged.

In conclusion, our data show that TRASP is highly immunogenic both for humoral and T cell responses and induces strong protection in mice and dogs. Although genetic vaccination confers robust protection, there are some counterpoints. During the COVID-19 pandemic, we learned that the industrial production of non-replicative adenovirus vaccines is not a main limitation. However, there are some practical issues that need to be considered, first, adenovirus 5 is a prevalent virus in human populations, and a vaccine using adenovirus 5 as a vector maybe less effective in seropositive patients. The same is to say about the adenovirus 26 and the chimpanzee (ChadOx), which were widely used in the COVID-19 vaccines. Secondly, the adenovirus vaccines were shown to have a more pronounced side effect, in particular in the first dose^64^. Furthermore, despite the production of adenovirus vaccines on an industrial scale is feasible, the costs of production of recombinant proteins in bacteria is cheaper, and this infrastructure is readily available in the middle-income Countries of Latin America. Hence, considering the quality of immune response, infrastructure requirement for its production in GMP conditions, the TRASP-pICLC formulation appears to be a better strategy for further development of a vaccine for Chagas’ disease.

## Methods

### Ethics statement

The experiments were carried out following the recommendations of the Guide for the Care and Use of Laboratory Animals of the Brazilian National Council of Animal Experimentation (CONCEA). The protocols for mouse and dog experiments were approved by Fundação Oswaldo Cruz and Universidade Federal de Ouro Preto Ethics Commission on Animal Use (CEUA), LW 02/19 and 2017/37, respectively.

### Mice, dogs, and parasites

Female C57BL/6 mice, 4–6 weeks old, were purchased from the Center for Laboratory Animal Facilities of the Federal University of Minas Gerais (CEBIO-UFMG). IFN-γ knockout (IFN-γ^−/−^), β-2 microglobulin knockout (β-2m^−/−^), and μMT (B KO), originally from Jax Laboratories, were bred at Fiocruz-Minas animal facilities. Bloodstream trypomastigotes of the Y strain of *T. cruzi* (DTU TcII) were obtained from mice infected 7 days earlier. Each mouse received 10^4^ trypomastigotes diluted in 0.1 ml phosphate buffered saline (PBS), administered intraperitoneally^12,13,19,23,26^. Alternatively, immunized mice were challenged with 5 × 10^3^ bloodstream trypomastigotes of Berenice-78 (DTU TcII) or CL-Brener (DTU TcVI)^65^.

In the canine experiments, we utilized 36 mongrel dogs 6 months old (15 males and 21 females) born in the kennel at UFOP, Minas Gerais, Brazil. They were treated with anthelminthic drugs and vaccinated against the most common canine infectious pathogens. Later, the animals were infected with blood trypomastigotes of Berenice-78 strain^66^ that were maintained in Swiss Webster mice. Each dog received a dose of 2000 trypomastigotes/kg body weight intraperitoneally.

### Epitope prediction

Three programs were used for epitope prediction: SYFPEITHI^67^; BIMAS^68^, and The Immune Epitope Database and Analysis Resource (IEDB)^69^. Potential HLA-ABC-binding epitopes were sought in the sequences of TS (accession Q4CZ79) and ASP-2 (accession U77951.1). The search was performed for all HLA alleles present in at least two of the three programs.

### Expression and purification of recombinant proteins

Plasmids pET-21a cloned with the sequences of TRASP, rTS, or ASP-2 plus a tag of six histidine residues were purchased from Genscript®. Each construction was transferred to Shuffle T7 Express *E. coli* for the production of the recombinant proteins. Transformed bacteria were grown at 37 °C in LB medium containing 100 µg/mL of kanamycin, and protein expression was induced by IPTG at 25 °C for sixteen hours. TRASP, rTS, and rASP-2 were purified from crude bacterial extracts by immobilized metal affinity chromatography, following the manufacturer’s protocols (Novagen, Inc.Madison, WI). Then, proteins were dialyzed for urea reduction to 1 M and endotoxins were removed through an ε-poly-l-lysine resin (Thermo Fisher). The levels of endotoxin were quantified with the kit ToxinSensor™ Chromogenic LAL Endotoxin Assay (GenScript), and it was achieved acceptable levels for inoculation (<20 EU/dose). Finally, the purified recombinant proteins were analyzed by SDS-PAGE through the visualization of bands corresponding to the estimated molecular weight (original uncropped gel is supplied in Supplementary Fig. 1). Two additional recombinant versions of TS, expressed in *E. coli*, one without the C-terminal SAPA-repeats (TS w/o SAPA) and another containing only the repetitive SAPA domain (SAPA) were purified using a his-tag and nickel column system and employed in our ELISA to detect antibodies.

### Amplification and purification of plasmids and adenovirus encoding ASP-2 or TS genes

The genetic vaccination (DNA TS/ASP + Ad TS/ASP) was performed with the plasmids p154/13 and pIgSPclone9, which are recombinant plasmids that encode the sequences of TS (DNA TS) and ASP-2 (DNA ASP-2), respectively. We also utilized human replication-deficient adenoviruses type 5 (Ad5) expressing TS (AdTS) or ASP-2 (AdASP-2). The constructs were generated as described previously in refs. ^19,70,71^.

Briefly, commercially available pcDNA3 (Invitrogen) was ligated with the sequences of signal peptide and the catalytic domain of the TS protein gene 154 (accession Q26964), originating the plasmid p154/13. The plasmid pIgSPclone9 was generated through the ligation of pcDNA3 with the sequences of immunoglobulin κ chain SP (IgSP) and ASP-2 protein clone 9 gene (accession AY186572). Both constructs were grown in *E. coli* DH5α and purified on cesium chloride density gradients.

Recombinant adenoviruses were generated through a cotransfection of the plasmids pJM17 (encodes the complete Ad5 genome) and pAdCMV-TS or pAdCMV-ASP-2 (bearing the sequences of TS or ASP-2 from p154/13 or pIgSPclone9, respectively)into HEK 293 cells. The assembled viruses AdTS and AdASP-2 were propagated in HEK 293 cells and purified in a cesium chloride isopycnic centrifugation.

### Immunizations

The purified protein TRASP was inoculated subcutaneously on the dorsal region in mice and on the right flank in dogs. Both species received three doses, at 21 days apart for mice and 30 days for dogs. In mice, administrations contained 10 μg of TRASP supplemented with 18 μg of CpG B344 (synthetized by Alpha DNA, Montreal, Canada) plus 30% (v/v) of Alum Rehydragel L.V. solution (Reheis, Berkeley Heights, NJ)^72,73^ or 50 μg of Hiltonol® (Poly-ICLC) supplied by Oncovir (Washington, D.C.)^28–41^. In dogs, each dose contained 150 μg of TRASP adjuvanted with 500 μg of Hiltonol® (Poly-ICLC).

The heterologous prime-boost protocol with plasmids and adenovirus for mice comprised of a prime dose with 50 μg of DNA TS plus 50 μg of DNA ASP-2 and a boost dose, twenty-one days later, containing 10^8^ PFU of AdTS plus 10^8^ PFU of AdASP-2. The control groups received 100 μg of pcDNA3 (DNA∅) and 2 × 10^8^ PFU of Ad5 expressing β-galactosidase (AdβGal).^19,23,26^. Dogs received a prime of 200 μg of DNA TS plus 200 μg of DNA ASP-2 and two boosts, each one containing 10^9^ PFU of AdTS plus 10^9^ PFU of AdASP-2. Each dose was inoculated intramuscularly at 21 days apart. Control groups received 400 μg of DNA∅ and two boosts with 2 × 10^9^ PFU of AdβGal.

### Detection of antigen-specific antibodies

For mice, ELISA plates were coated with 1 μg/well of rASP-2, rTS or TRASP, reacted with sera diluted 1:200, followed by incubation with secondary antibodies anti-total IgG, anti-IgG1, or anti-IgG2c conjugated with streptavidin-HRP (Cat 1030-05, 1070-05, 1079-05, Southern Biotech), all diluted 1:5000. For dogs, we used ELISA plates (Costar) coated with 100 ng/well of *T. cruzi* recombinant antigens, sera diluted 1:100 and anti-total IgG, IgG1, IgG2, and IgM conjugated with streptavidin-HRP (Cat A40-123P, A40-120P, A40121P, BEYA40-116P, Bethyl Laboratories, INC), all diluted 1:100,000. The reactions were developed with the substrate 3,3’ 5,5’-tetrametylbenzidine (TMB) from SIGMA and read at 450 nm^19,73^.

### Complement-mediated lysis assay

The assay was conducted as previously described in refs. ^74,75^. First, antibody-free trypomastigotes were isolated from infected B lymphocyte-deficient mice (B KO). At 9 DPI, *T. cruzi*-infected B KO mice were bled, and the blood was centrifuged at 5000 rpm for 15 min to collect the plasma. The parasites were then purified by centrifugation at 1000×*g* for 20 min, counted and adjusted to 10^6^ trypomastigotes/mL in PBS 10% SFB. Twenty-five microliters of antibody-free trypomastigotes was added to 100 μL of serum from TRASP-immunized mice and incubated at 37 °C for 30 min. Serum from *T. cruzi*-infected mice at 15 DPI was used as a positive control. Then, 25 μL of the mixture was incubated with 25 μL of complement-rich fresh human serum (HuS) or inactivated (iHuS) at 37 °C for 30 min. After that, motile parasites were put on ice and counted in a Neubauer chamber. The percentile of lysis was calculated as follows:

$$\% \,{lysis}=100-\frac{{number}\,{of}\,{parasites}\,{after}\,{incubation}\,{with}\,{HuS}}{{number}\,{of}\,{parasites}\,{after}\,{incubation}\,{with}\,{iHuS}}\,x\,100\,$$

### Lymphocyte proliferation and cytokine measurements

Mouse splenocytes were isolated by macerating the spleen through a 100 μm pore cell strainer (Cell Strainer, BD Falcon) followed by treatment with ACK buffer for erythrocytes lysis. The number of cells was adjusted to 10^6^ cells per well and then stimulated with 10 μg/mL of rTS, rASP-2, or TRASP. Concanavalin A (Sigma, 5 μg/mL) was used as a positive control. The supernatants were collected 48 h post-stimulation and the levels of IFN-γ and IL-10 determined by ELISA (R&D Systems)^19,73^.

Blood samples from dogs were collected from the jugular vein and peripheral blood mononuclear cells (PBMCs) purified by Ficoll-Hypaque density gradient (Histopaque^®^ 1.077; Sigma). The PBMCs were suspended in RPMI at 10^7^ cells/mL, stained with 10 μM of succinimidyl ester of fluorescein carboxy diacetate (CFSE, Molecular Probes) and cultured for 5 days in the presence of total *T. cruzi* extract of Be-78 (Ag Be-78), rASP-2, rTS, or TRASP at a final concentration of 10 μg/mL. Concanavalin A (Sigma) at 4 μg/mL was a positive control. After the incubation period, the supernatant was collected and stored at −80 °C for detection of IFN-γ and IL-10 by ELISA (R&D Systems). The PBMCs were collected, labeled with anti-CD4 PE (YKIX302.9, Bio-Rad) and anti-CD8 Alexa Fluor 647 (YCATE55.9, Bio-Rad), and for the proliferation assay, a total of 50,000 events were read on a BD FACScalibur flow cytometer.

### Flow cytometry

For immunophenotyping splenocytes derived from immunized mice^76^, a total of 2 × 10^6^ cells were incubated for 18 h at 37 °C and 5% CO_2_ with RPMI 1640 medium alone or containing 10 μg/mL of TRASP. During the last 6 h of culture, GolgiStop and GolgiPlug Protein Transport Inhibitors (BD Biosciences) were added to the cell cultures. The splenocytes were then washed with PBS, stained with Live/Dead reagent (Invitrogen), and incubated with FcBlock (BD Biosciences). The following mAbs were used to label cell surface markers: anti-CD3 PE-Cy5 or APC-Cy7 (145-2C11, BD), anti-CD4 Alexa Fluor 700 (RM4-5, eBioscience), anti-CD8 Alexa Fluor 700 (53-6.7, BD), anti-CD62L APC (MEL-14, BD), anti-CD44 BV 605 (IM7, eBioscience), anti-CD278/ICOS FITC (7E-1769, BD), anti-PD-1 PE-Texas Red (J43, Invitrogen), anti-CXCR3 Pacific Blue (CXCR3-173, BD), anti-CD19 FITC (1D3, eBioscience), anti-CD27 PE (LG.7F9, eBioscience), and anti-IgD Pacific Blue (11-26, BD). For intracellular staining, cells were washed, fixed, and permeabilized according to the manufacturer’s instructions (Cytofix/Cytoperm, BD Biosciences or Foxp3/Transcription Factor Staining, eBioscience) and stained with anti-IFN-γ PerCP-Cy5.5 or APC (XMG1.2, eBioscience) and anti-Bcl-6 Pacific Blue (K112-91, BD). Flow cytometry was carried out using a BD LSRFortessa and ~100,000 live CD3^+^ CD4^+^ or CD3^+^ CD8^+^ cells were acquired. Data were analyzed using FlowJo software.

For experiments with dogs, the blood was collected and then incubated with RPMI with PMA (25 ng/mL), ionomycin (1 μg/mL), and brefeldin A (10 μg/mL, Sigma). For comparison, there were also tubes containing only RPMI and brefeldin A. The cells were then washed and stained with anti-CD8 Alexa Fluor 647 (YCATE55.9, Bio-Rad) and anti-CD4 FITC (YKIX302.9, Bio-Rad). For intracellular staining, cells were permeabilized and stained with anti-IFN-γ PE (CC302, Bio-Rad). The samples were read on the FACSCalibur flow cytometer in which 100,000 events were acquired. The final data are represented by indexes, determined by the percentage of positive cells in the PMA/Ionomycin-stimulated cells divided by the paired unstimulated control.

### Quantification of parasite load by qPCR

Fragments of the heart were collected during necropsy at 180 days after vaccination, then subjected to procedures of DNA extraction using WizardTM Genomic DNA Purification Kit (Promega, Madison, WI, USA), following manufacturer’s recommendations. The qPCR was carried out with the Platinum SYBR Green protocol (Thermo Fisher) and the parasite load was quantified by the standard curve method. The 195-bp repeat DNA-specific primers TCZ-F 5′-GCTCTTGCCCACAMGGGTGC-3′, where M = A or C and TCZ-R 5′-CCAAGCAGCGGATAGTTCAGG-3′ were used for quantifying the *T. cruzi* DNA. As an endogenous mouse gene we used the TNF-5241 5′-TCCCTCTCATCAGTTCTATGGCCCA-3′ and TNF-5411 5′-CAGCAAGCATCTATGCACTTAGACCCC-3′^77^.

### Histopathology

For histopathological analysis of myocardial tissues, dogs were necropsied at 180 days after vaccination and fragments of the heart were fixed in 4% buffered formalin (pH 7.2) for 48 h and embedded in paraffin. Sections (4 mm thick) were mounted on glass slides and stained with Haematoxylin-Eosin (HE) for quantification of the inflammatory process. Morphometric studies of inflammation involved analyzing images of 25 randomly selected fields (total area 1.1 × 10^6^ μm^2^) of left ventricles sections on a single slide per animal. Inflammatory infiltration was quantified by counting the cell nuclei present in the sections. Sections were viewed with a 40x objective and images were digitized by microcamera AxioCam MRc (Zeiss) associated with Zeiss microscopy Axio Imager Z2; all images were analyzed using the image processing and analysis software Leica Qwin V3 (Leica Microsystems, Wetzlar, Germany) at Multiuser Laboratory of Núcleo de Pesquisas em Ciências Biológicas of UFOP.

### Statistical analysis

Statistical analysis was conducted using GraphPad Prism 6.0 for Mac (GraphPad Inc, USA). First, outliers were detected with Grubbs’s test and then D’Agostino–Pearson was run to verify data normality. The tests used on each data analysis are explained in figure legends. In general, comparison between the groups was performed through two-way ANOVA and Tukey’s multiple comparisons test, or Friedman test and Dunns’ multiple comparisons test, depending on data distribution. For survival analysis, the log-rank test was used. Statistical differences were considered significant when *p* values ≤0.05.

### Reporting summary

Further information on research design is available in the Nature Research Reporting Summary linked to this article.

## Supplementary information

Supplementary Information

Reporting Summary

## Data Availability

The authors declare that all data supporting the findings of this study are available within the paper and its supplementary information files. If any more information is needed, data are available from the corresponding author upon reasonable request. For epitope prediction of *Trans*-sialidase (accession Q4CZ79) and ASP-2 (accession U77951.1) proteins, we used The Immune Epitope Database (IEDB), Bimas, and SYFPEITHI.

## References

[1] Rassi A, Rassi A, Marin-Neto JA (2010). Chagas disease. Lancet.

[2] Lee BY, Bacon KM, Bottazzi ME, Hotez PJ (2013). Global economic burden of Chagas disease: a computational simulation model. Lancet Infect. Dis..

[3] Franco-Paredes C (2020). A deadly feast: elucidating the burden of orally acquired acute Chagas disease in Latin America - Public health and travel medicine importance. Travel Med. Infect. Dis..

[4] Garcia MN, Woc-Colburn L, Aguilar D, Hotez PJ, Murray KO (2015). Historical perspectives on the epidemiology of human Chagas disease in Texas and recommendations for enhanced understanding of clinical chagas disease in the Southern United States. PLoS Negl. Trop. Dis..

[5] Garcia MN (2015). Evidence of autochthonous Chagas disease in southeastern Texas. Am. J. Trop. Med. Hyg..

[6] Bartsch SM (2020). The potential economic value of a therapeutic Chagas disease vaccine for pregnant women to prevent congenital transmission. Vaccine.

[7] Franco-Paredes C, Bottazzi ME, Hotez PJ (2009). The unfinished public health agenda of chagas disease in the era of globalization. PLoS Negl. Trop. Dis..

[8] Filardi LS, Brener Z (1987). Susceptibility and natural resistance of *Trypanosoma cruzi* strains to drugs used clinically in Chagas disease. Trans. R Soc. Trop. Med. Hyg..

[9] Pinazo MJ (2013). Benznidazole-related adverse drug reactions and their relationship to serum drug concentrations in patients with chronic chagas disease. Antimicrob. Agents Chemother..

[10] Araujo AF (2005). CD8+-T-cell-dependent control of *Trypanosoma cruzi* infection in a highly susceptible mouse strain after immunization with recombinant proteins based on amastigote surface protein 2. Infect. Immun..

[11] Araujo AF (2014). Genetic vaccination against experimental infection with myotropic parasite strains of *Trypanosoma cruzi*. Mediators Inflamm..

[12] Barbosa RP (2013). Vaccination using recombinants influenza and adenoviruses encoding amastigote surface protein-2 are highly effective on protection against *Trypanosoma cruzi* infection. PLoS ONE.

[13] de Alencar BC, Araujo AF, Penido ML, Gazzinelli RT, Rodrigues MM (2007). Cross-priming of long lived protective CD8+ T cells against *Trypanosoma cruzi* infection: importance of a TLR9 agonist and CD4+ T cells. Vaccine.

[14] de Alencar BC (2009). Perforin and gamma interferon expression are required for CD4+ and CD8+ T-cell-dependent protective immunity against a human parasite, Trypanosoma cruzi, elicited by heterologous plasmid DNA prime-recombinant adenovirus 5 boost vaccination. Infect. Immun..

[15] Dominguez MR (2011). Subdominant/cryptic CD8 T cell epitopes contribute to resistance against experimental infection with a human protozoan parasite. PLoS ONE.

[16] Ersching J (2016). The Combined deficiency of immunoproteasome subunits affects both the magnitude and quality of pathogen- and genetic vaccination-induced CD8+ T cell responses to the human protozoan parasite *Trypanosoma cruzi*. PLoS Pathog..

[17] Ferreira CP (2017). LFA-1 mediates cytotoxicity and tissue migration of specific CD8(+) T cells after heterologous prime-boost vaccination against *Trypanosoma cruzi* infection. Front. Immunol..

[18] Haolla FA (2009). Strain-specific protective immunity following vaccination against experimental *Trypanosoma cruzi* infection. Vaccine.

[19] Machado AV (2006). Long-term protective immunity induced against *Trypanosoma cruzi* infection after vaccination with recombinant adenoviruses encoding amastigote surface protein-2 and trans-sialidase. Hum. Gene Ther..

[20] Pereira IR (2015). A human type 5 adenovirus-based *Trypanosoma cruzi* therapeutic vaccine re-programs immune response and reverses chronic cardiomyopathy. PLoS Pathog..

[21] Rigato PO (2011). Heterologous plasmid DNA prime-recombinant human adenovirus 5 boost vaccination generates a stable pool of protective long-lived CD8(+) T effector memory cells specific for a human parasite, *Trypanosoma cruzi*. Infect. Immun..

[22] Silverio JC (2012). CD8+ T-cells expressing interferon gamma or perforin play antagonistic roles in heart injury in experimental *Trypanosoma cruzi*-elicited cardiomyopathy. PLoS Pathog..

[23] Tzelepis F (2008). Infection with Trypanosoma cruzi restricts the repertoire of parasite-specific CD8+ T cells leading to immunodominance. J. Immunol..

[24] Tzelepis F (2006). Distinct kinetics of effector CD8+ cytotoxic T cells after infection with *Trypanosoma cruzi* in naive or vaccinated mice. Infect. Immun..

[25] Vasconcelos JR (2012). Pathogen-induced proapoptotic phenotype and high CD95 (Fas) expression accompany a suboptimal CD8+ T-cell response: reversal by adenoviral vaccine. PLoS Pathog..

[26] Vasconcelos JR (2014). Adenovirus vector-induced CD8(+) T effector memory cell differentiation and recirculation, but not proliferation, are important for protective immunity against experimental *Trypanosoma cruzi* Infection. Hum. Gene Ther..

[27] Vasconcelos JR (2004). Protective immunity against *Trypanosoma cruzi* infection in a highly susceptible mouse strain after vaccination with genes encoding the amastigote surface protein-2 and trans-sialidase. Hum. Gene Ther..

[28] Martins KA, Bavari S, Salazar AM (2015). Vaccine adjuvant uses of poly-IC and derivatives. Expert Rev. Vaccines.

[29] Sultan H, Salazar AM, Celis E (2020). Poly-ICLC, a multi-functional immune modulator for treating cancer. Semin. Immunol..

[30] Butowski N (2009). A phase II clinical trial of poly-ICLC with radiation for adult patients with newly diagnosed supratentorial glioblastoma: a North American Brain Tumor Consortium (NABTC01-05). J. Neurooncol..

[31] Diaz-San Segundo F (2014). Poly ICLC increases the potency of a replication-defective human adenovirus vectored foot-and-mouth disease vaccine. Virology.

[32] Hartman LL (2014). Pediatric phase II trials of poly-ICLC in the management of newly diagnosed and recurrent brain tumors. J. Pediatr. Hematol. Oncol..

[33] Kende M, Paragas J, Salazar AM (2019). The efficacy of poly-ICLC against Ebola-Zaire virus (EBOV) infection in mice and cynomolgus monkeys. Antiviral Res..

[34] Kyi C (2018). Therapeutic immune modulation against solid cancers with intratumoral poly-ICLC: a pilot trial. Clin. Cancer Res..

[35] Liu H (2018). WT1 peptide vaccine in Montanide in contrast to poly ICLC, is able to induce WT1-specific immune response with TCR clonal enrichment in myeloid leukemia. Exp. Hematol. Oncol..

[36] Okada H (2015). Induction of robust type-I CD8+ T-cell responses in WHO grade 2 low-grade glioma patients receiving peptide-based vaccines in combination with poly-ICLC. Clin. Cancer Res..

[37] Pavlick A (2020). Combined vaccination with NY-ESO-1 protein, poly-ICLC, and montanide improves humoral and cellular immune responses in patients with high-risk melanoma. Cancer Immunol. Res..

[38] Rosenfeld MR (2010). A multi-institution phase II study of poly-ICLC and radiotherapy with concurrent and adjuvant temozolomide in adults with newly diagnosed glioblastoma. Neuro Oncol.

[39] Sabbatini P (2012). Phase I trial of overlapping long peptides from a tumor self-antigen and poly-ICLC shows rapid induction of integrated immune response in ovarian cancer patients. Clin. Cancer Res..

[40] Saxena M (2019). Poly-ICLC, a TLR3 agonist, induces transient innate immune responses in patients with treated HIV-infection: a randomized double-blinded placebo controlled trial. Front. Immunol..

[41] Zhu X (2007). Toll like receptor-3 ligand poly-ICLC promotes the efficacy of peripheral vaccinations with tumor antigen-derived peptide epitopes in murine CNS tumor models. J. Transl. Med..

[42] Reis-Cunha JL (2022). Accessing the variability of multicopy genes in complex genomes using unassembled next-generation sequencing reads: the case of *Trypanosoma cruzi* multigene families. mBio.

[43] Anselmi A, Gurdiel O, Suarez JA, Anselmi G (1967). Disturbances in the A-V conduction system in Chagas’ myocarditis in the dog. Circ. Res..

[44] Lana M (1991). Humoral immune response in dogs experimentally infected with *Trypanosoma cruzi*. Mem. Inst. Oswaldo Cruz.

[45] Hartley AN, Cooley G, Gwyn S, Orozco MM, Tarleton RL (2014). Frequency of IFNgamma-producing T cells correlates with seroreactivity and activated T cells during canine *Trypanosoma cruzi* infection. Vet. Res..

[46] Guedes PM (2009). Development of chronic cardiomyopathy in canine Chagas disease correlates with high IFN-gamma, TNF-alpha, and low IL-10 production during the acute infection phase. Vet. Immunol. Immunopathol..

[47] Pitcovsky TA (2001). Epitope mapping of trans-sialidase from *Trypanosoma cruzi* reveals the presence of several cross-reactive determinants. Infect. Immun..

[48] Almeida IC, Ferguson MA, Schenkman S, Travassos LR (1994). Lytic anti-alpha-galactosyl antibodies from patients with chronic Chagas’ disease recognize novel O-linked oligosaccharides on mucin-like glycosyl-phosphatidylinositol-anchored glycoproteins of *Trypanosoma cruzi*. Biochem. J..

[49] Dc-Rubin SS, Schenkman S (2012). T rypanosoma cruzi trans-sialidase as a multifunctional enzyme in Chagas’ disease. Cell Microbiol..

[50] Freitas LM (2011). Genomic analyses, gene expression and antigenic profile of the trans-sialidase superfamily of Trypanosoma cruzi reveal an undetected level of complexity. PLoS ONE.

[51] Junqueira C (2010). The endless race between *Trypanosoma cruzi* and host immunity: lessons for and beyond Chagas disease. Expert Rev. Mol. Med..

[52] Chadalawada S (2020). Risk of chronic cardiomyopathy among patients with the acute phase or indeterminate form of Chagas disease: a systematic review and meta-analysis. JAMA Netw. Open.

[53] Cunha-Neto E, Teixeira PC, Nogueira LG, Kalil J (2011). Autoimmunity. Adv. Parasitol..

[54] Gutierrez FR, Guedes PM, Gazzinelli RT, Silva JS (2009). The role of parasite persistence in pathogenesis of Chagas heart disease. Parasite Immunol..

[55] Burle-Caldas GA (2022). Disruption of active trans-sialidase genes impairs egress from mammalian host cells and generates highly attenuated *Trypanosoma cruzi* parasites. mBio.

[56] Bivona AE, Alberti AS, Cerny N, Trinitario SN, Malchiodi EL (2020). Chagas disease vaccine design: the search for an efficient *Trypanosoma cruzi* immune-mediated control. Biochim. Biophys. Acta Mol. Basis Dis..

[57] Rios LE, Vazquez-Chagoyan JC, Pacheco AO, Zago MP, Garg NJ (2019). Immunity and vaccine development efforts against *Trypanosoma cruzi*. Acta Trop..

[58] Belew AT (2017). Comparative transcriptome profiling of virulent and non-virulent *Trypanosoma cruzi* underlines the role of surface proteins during infection. PLoS Pathog..

[59] Dos Santos LI (2015). Blockade of CTLA-4 promotes the development of effector CD8+ T lymphocytes and the therapeutic effect of vaccination with an attenuated protozoan expressing NY-ESO-1. Cancer Immunol. Immunother..

[60] Junqueira C (2011). *Trypanosoma cruzi* as an effective cancer antigen delivery vector. Proc. Natl Acad. Sci. USA.

[61] Sanchez Alberti A (2017). Engineered trivalent immunogen adjuvanted with a STING agonist confers protection against *Trypanosoma cruzi* infection. NPJ Vaccines.

[62] Dotiwala F (2016). Killer lymphocytes use granulysin, perforin and granzymes to kill intracellular parasites. Nat. Med..

[63] Tarleton RL (2015). CD8+ T cells in *Trypanosoma cruzi* infection. Semin. Immunopathol..

[64] Folegatti PM (2020). Safety and immunogenicity of the ChAdOx1 nCoV-19 vaccine against SARS-CoV-2: a preliminary report of a phase 1/2, single-blind, randomised controlled trial. Lancet.

[65] Zingales B (2009). A new consensus for *Trypanosoma cruzi* intraspecific nomenclature: second revision meeting recommends TcI to TcVI. Mem. Inst. Oswaldo Cruz.

[66] Lana, M. & Chiari, C. A. [Comparative biological characterization of Berenice and Berenice-78 strains of *Trypanosoma cruzi* isolated from the same patient at different times]. *Mem. Inst. Oswaldo Cruz* **81**, 247–253 (1986). 10.1590/s0074-02761986000300001 3106753

[67] Rammensee H, Bachmann J, Emmerich NP, Bachor OA, Stevanovic S (1999). SYFPEITHI: database for MHC ligands and peptide motifs. Immunogenetics.

[68] Parker KC, Bednarek MA, Coligan JE (1994). Scheme for ranking potential HLA-A2 binding peptides based on independent binding of individual peptide side-chains. J. Immunol..

[69] Vita R (2019). The immune epitope database (IEDB): 2018 update. Nucleic Acids Res.

[70] Costa F (1998). Immunization with a plasmid DNA containing the gene of trans-sialidase reduces *Trypanosoma cruzi* infection in mice. Vaccine.

[71] Boscardin SB, Kinoshita SS, Fujimura AE, Rodrigues MM (2003). Immunization with cDNA expressed by amastigotes of *Trypanosoma cruzi* elicits protective immune response against experimental infection. Infect. Immun..

[72] Bartholomeu DC (2008). Recruitment and endo-lysosomal activation of TLR9 in dendritic cells infected with *Trypanosoma cruzi*. J. Immunol..

[73] Junqueira C (2012). *Trypanosoma cruzi* adjuvants potentiate T cell-mediated immunity induced by a NY-ESO-1 based antitumor vaccine. PLoS ONE.

[74] Krettli AU, Weisz-Carrington P, Nussenzweig RS (1979). Membrane-bound antibodies to bloodstream *Trypanosoma cruzi* in mice: strain differences in susceptibility to complement-mediated lysis. Clin. Exp. Immunol..

[75] Takehara HA, Cardoso DF, da Silva AM, Mota I (1988). Lytic antibodies elicited by *Trypanosoma cruzi* infection recognize epitopes present on both bloodstream trypomastigote and epimastigote forms of parasite. Rev. Inst. Med. Trop. Sao Paulo.

[76] Cossarizza, A. et al. Guidelines for the use of flow cytometry and cell sorting in immunological studies (second edition). *Eur. J. Immunol*. **49**, 1457–1973 (2019). 10.1002/eji.201970107 PMC7350392 31633216

[77] Cummings KL, Tarleton RL (2003). Rapid quantitation of *Trypanosoma cruzi* in host tissue by real-time PCR. Mol. Biochem. Parasitol..

