# Supplementary material for: ASP-2/Trans-sialidase chimeric protein induces robust protective immunity in experimental models of Chagas’ disease
Source: NPJ Vaccines. 2023 May 31;8:81. doi: 10.1038/s41541-023-00676-0 (PMC10231858; doi:10.1038/s41541-023-00676-0)
Supplement: Supplementary file 1 — Supplementary Information [file 41541_2023_676_MOESM1_ESM.pdf]

Supplementary Figure 1

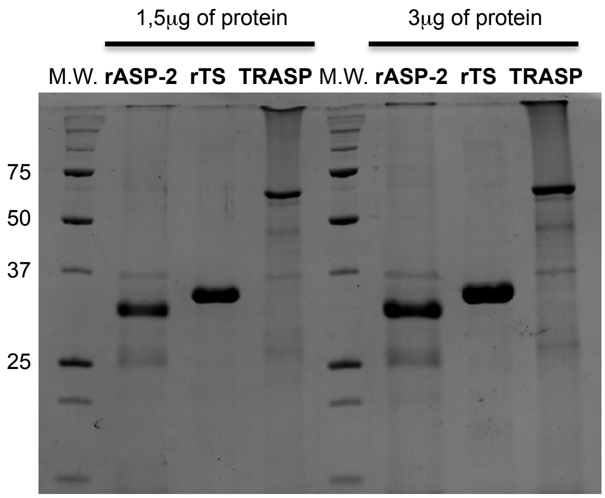

Supplementary Figure 1. Original uncropped gel from Figure 1b. The recombinant proteins rASP-2, rTS and TRASP were purified and analyzed by SDS-PAGE.

Supplementary Figure 2

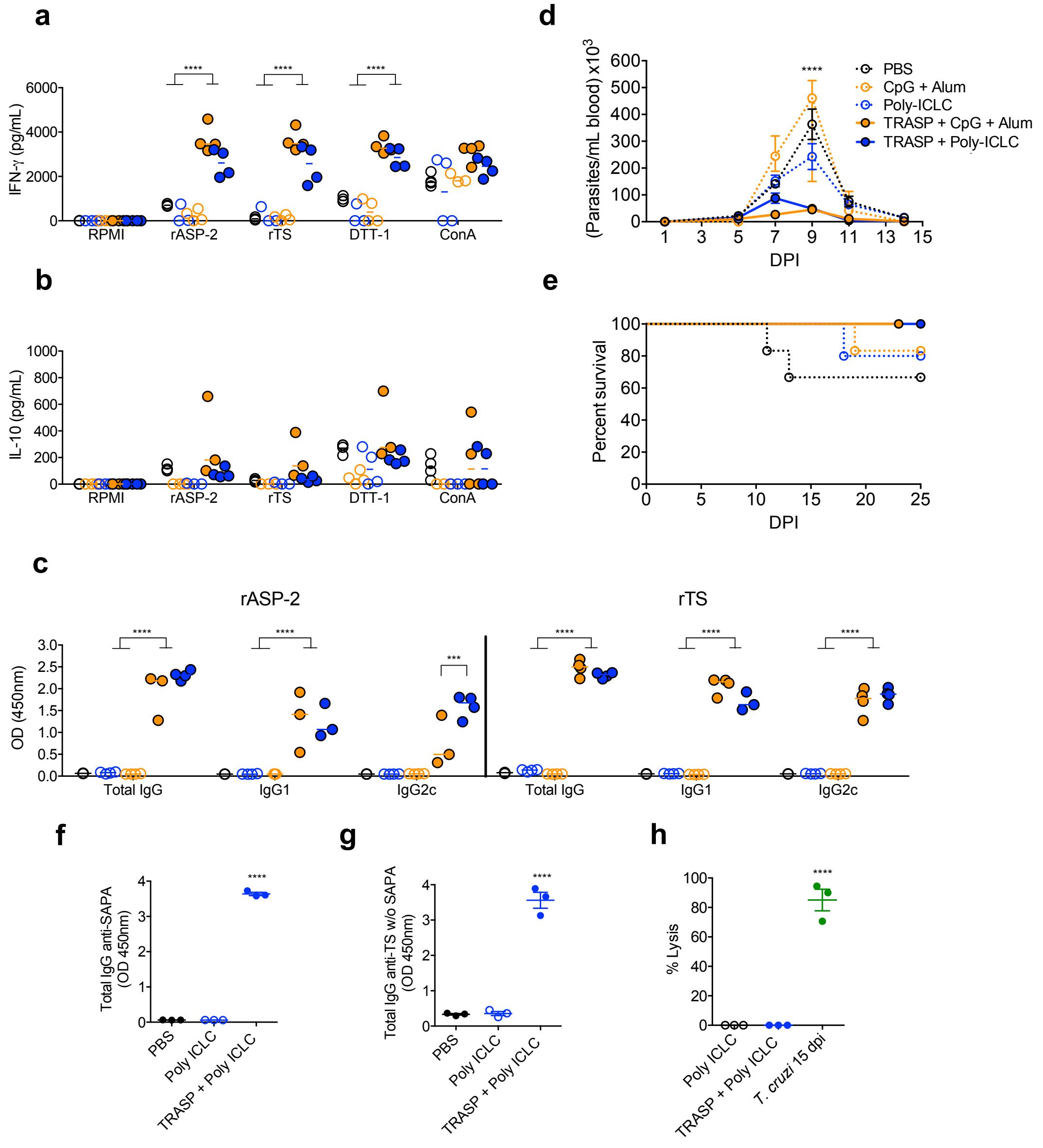

Supplementary Figure 2. Comparison of the efficacy of TRASP associated with different adjuvants and antibodies measurements. Thirty days after the boost, the cellular immune response was assessed through the production of IFN-g **(a)** and IL-10 **(b)** by splenocytes from immunized mice under specific stimuli. Antibodies total IgG, IgG1 and IgG2c anti-ASP-2 and anti-TS were dosed in mice sera **(c)**. Additionally, part of immunized animals was challenged with 10^4 trypomastigotes of T. cruzi Y strain, and then parasitemia **(d)** and survival **(e)** were followed for 15 and 25 days, respectively. **f, g,** Levels of IgG anti-SAPA repeats **(f)** and anti-TS without SAPA **(g)**. **h,** Analysis of trypanolytic antibodies in the sera from control, immunized or infected mice. The statistical analysis was carried out using two-way ANOVA **(a-d)** or one-way ANOVA **(f-h)** followed by Tukey's multiple comparisons test. Data are representative of two independent experiments. **d,** Mean ± SEM (n = 4-6 mice per group). **** $p < 0.0001$.

Supplementary Figure 3

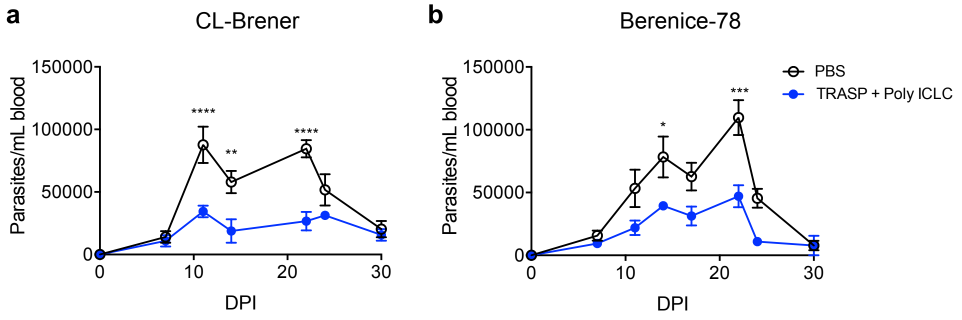

Supplementary Figure 3. Immunization with TRASP induces cross-protection. TRASP-immunized mice were infected with 5 x 10^3 blood trypomastigotes of CL-Brener **(a)** or Be-78 **(b)** and the levels of parasitemia were measured for 30 days. The statistical analysis was carried out using two-way ANOVA followed by Tukey's multiple comparisons test. Data are representative of two independent experiments, mean ± SEM (n = 6 mice per group). * $p < 0.05$; ** $p < 0.01$; *** $p < 0.001$; **** $p < 0.0001$.

Supplementary Figure 4

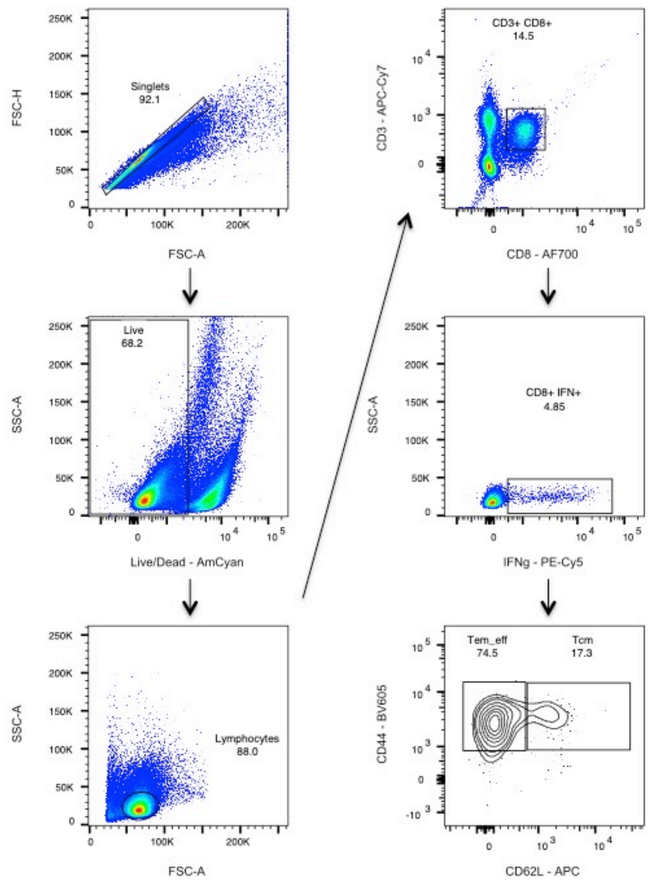

Supplementary Figure 4. Gating strategy of flow cytometry for characterization of IFN-γ-producing CD8⁺ T cells. Ninety days post vaccination, TRASP-immunized mice had their spleen harvested. The cells were isolated, stimulated with TRASP and then stained for memory CD8⁺ T cell characterization. Splenocytes were gated for singlets

(FSC-H X FSC-A), live (SSC-A x Live/Dead), lymphocytes (SSC-A x FSC-A), $CD3^+$ $CD8^+$ (CD3 x CD8), IFN-γ+ (SSC-A x IFN-γ) and with CD44 x CD62L they were separated into effector/effector memory ($CD44^+$ $CD62L^-$) and central memory ($CD44^+$ $CD62L^+$).

Supplementary Figure 5

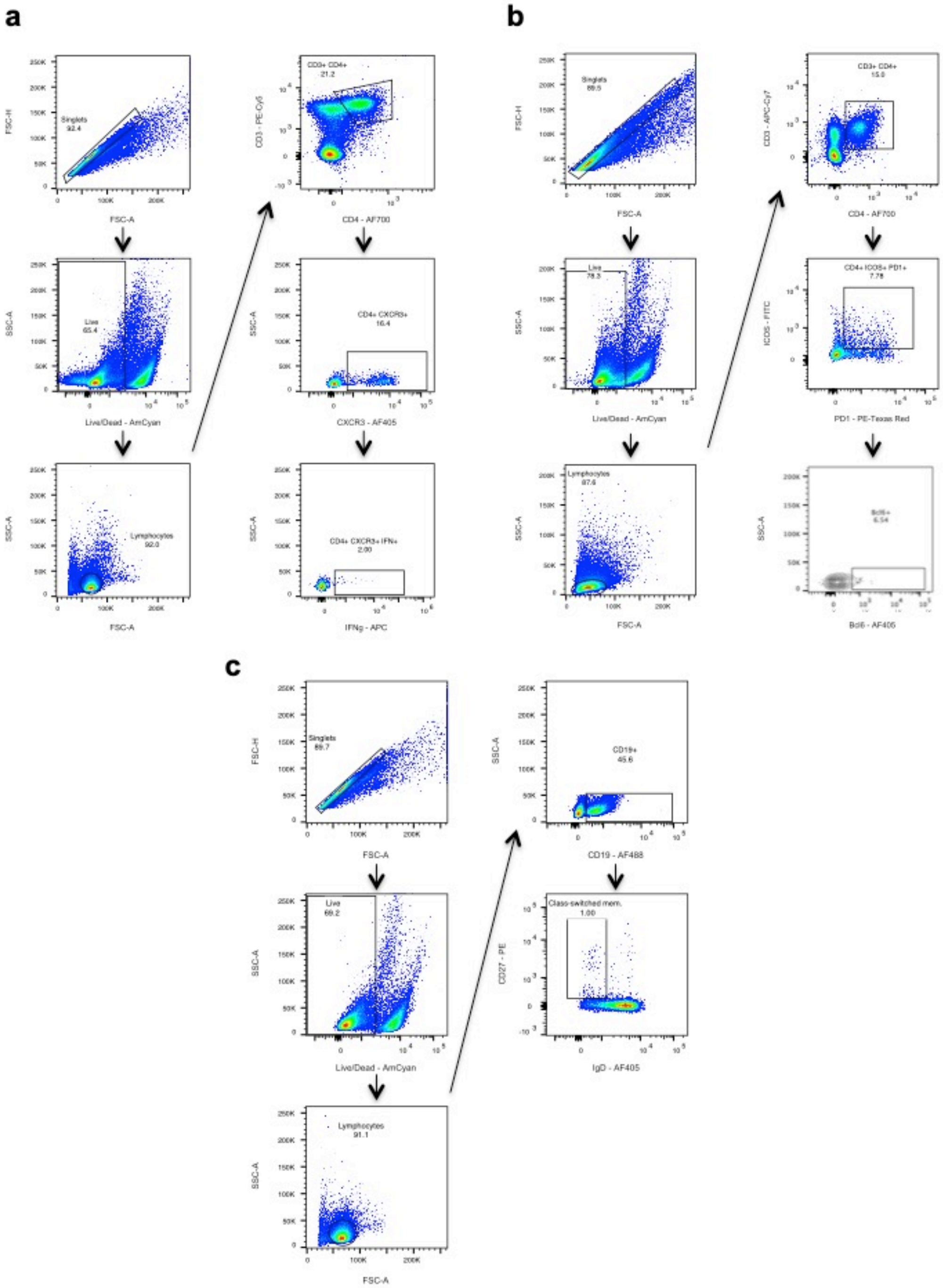

Supplementary Figure 5. Gating strategy of flow cytometry for characterization of $CD4^+$ T and B lymphocytes in the spleen. a,b, Splenocytes were gated for singlets (FSC-H X FSC-A), live (SSC-A x Live/Dead), lymphocytes (SSC-A x FSC-A), $CD3^+$ $CD4^+$ (CD3 x CD8) and **a,** $CXCR3^+$ (SSC-A x CXCR3) and IFN-γ+ (SSC-A x IFN-γ) for $CD4^+$ Th1 or **b,** $ICOS^+$ PD-1^+ (ICOS X PD-1) and $Bcl6^+$ (SSC-A x $Bcl6^+$) for $CD4^+$ Tfh. **c,** For class-switched memory B lymphocytes, cells were gated for singlets (FSC-H X FSC-A), live (SSC-A x Live/Dead), lymphocytes (SSC-A x FSC-A), $CD19^+$ (SSC-A x CD19) and IgD^- $CD27^+$ (IgD x CD27).

Supplementary Figure 6

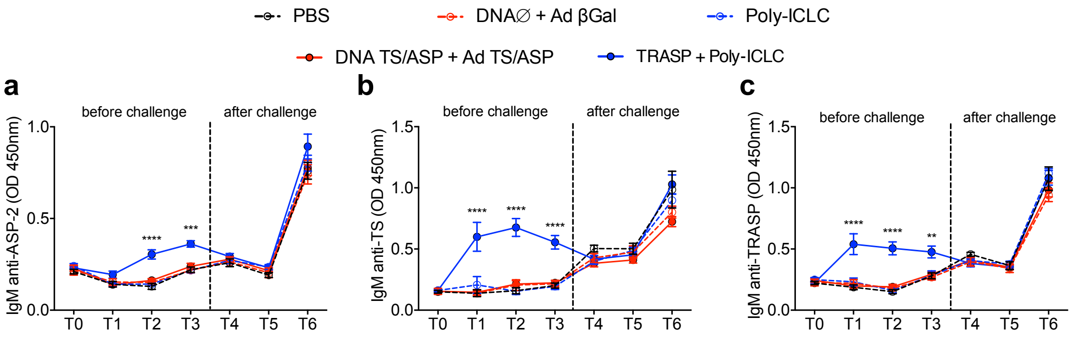

Supplementary Figure 6. IgM production induced by TRASP immunization. a-c, Specific IgM antibodies anti-ASP-2 **(a)**, anti-TS **(b)** and anti-TRASP **(c)** were measured in dogs' sera in different time points (T0-T6). The statistical analysis was carried out using two-way ANOVA and Tukey's multiple comparisons test. Mean ± SEM. ** $p < 0.01$; *** $p < 0.001$; **** $p < 0.0001$.

Supplementary Table 1

	Peptide	HLA-ABC	Percentile Rank (IEDB)	Conservation
Trans-sialidase	VSIGDENSAY	A* 01:01; A* 26:01; A* 29:02 A* 30:02; B* 15:01; B* 35:01; B* 46:01; B* 58:01	0.23; 0.79; 0.73; 0.28; 0.4; 0.86; 0.33; 0.92	Brazil, CL-Brener (Esmeraldo-like), Dm28c, Sylvio
	YRFANHAFTL	A* 24:02; B* 14:02; B* 27:05; B* 38:01; B* 39:01; B* 48:01	0.93; 0,21; 0.09; 0.29; 0.14; 0.49	Brazil, Dm28c, Sylvio
	HEAPRDATPL	B* 39:01; B* 40:01; B* 40:02; B* 44:02; B* 44:03; B* 48:01	0.94; 0.14; 0.4; 0.87; 0.86; 0.79	Y
	AERVPNGLKF	B* 18:01; B* 40:01; B* 40:02; B* 44:02; B* 44:03; B* 27:05	0.56; 0.23; 0.28; 0.02; 0.02; 0.08	Y
	RWLRDRLNLW	A* 23:01; A* 24:02; A* 32:01; B* 57:01; B* 58:01	0.22; 0.23; 0.19; 0.46; 0.93	Brazil, Dm28c, Sylvio, Y
	QRYRFANHAF	A* 23:01; A* 24:02; B* 14:02; B* 27:05; B* 38:01	0.81; 0.71; 0.81; 0,12; 0.91	Brazil, Dm28c, Sylvio
	VSQQGQNQRY	A* 01:01; A* 29:02; A* 30:02; B* 15:01; B* 58:01	0.11; 0.71; 0.14; 0.18; 0.83	Brazil, CL-Brener (Esmeraldo-like), Dm28c, Sylvio, Y
	SVLYKDDKLY	A* 01:01; A* 26:01; A* 29:02 A* 30:02; B* 15:01	0.8; 0.65; 0.47; 0.26; 0.4	Brazil, CL-Brener (Esmeraldo-like), Dm28c, Sylvio, Y
	GELRIIKSVL	B* 40:01; B* 40:02; B* 44:02; B* 44:03; B* 48:01	0.21; 0.21; 0.67; 0.68; 0.75	Brazil, Sylvio, Y
	VMLFTHPLNF	A* 23:01; A* 29:02; A* 32:01; B* 15:01; B* 46:01	0.41; 0.85; 0.22; 0.47; 0.58	Brazil, CL-Brener (Esmeraldo-like) Dm28c, Sylvio, Y
	RIIKSVLQSW	A* 25:01; A* 32:01; B* 53:01; B* 57:01; B* 58:01	0.3; 0.01; 0.92; 0.05; 0.07	Brazil, Y
	ENSAYSSVLY	A* 01:01; A* 25:01; A* 26:01; A* 29:02	0.14; 0.42; 0.17; 0.95	Brazil, CL-Brener (Esmeraldo-like), Dm28c, Sylvio
	MLFTHPLNFK	A* 03:01; A* 11:01; A* 30:01; A* 68:01	0.02; 0.22; 0.61; 0.96	Brazil, CL-Brener (Esmeraldo-like), Dm28c, Sylvio, Y
	EAPRDATPLL	A* 68:02; B* 07:02; B* 35:03; B* 51:01;	0.76; 0.18; 0.69; 0.8	-
	LHEINTNEVY	B* 18:01; B* 38:01; B* 44:02; B* 44:03	0.32; 0.7; 0.65; 0.49	Brazil, CL-Brener (Esmeraldo-like)
	SAHGTPSTTV	A* 68:02; B* 38:01; B* 39:01; B* 51:01	0.74; 0.4; 0.71; 0.95	-
	FAGVGGGALW	A* 25:01; B* 53:01; B* 57:01; B* 58:01;	0.28; 0.19; 0.32; 0.16	CL-Brener (Esmeraldo-like), Dm28c, Sylvio, Y
	EVYSLVFARL	A* 25:01; A* 26:01; A* 68:01; A* 68:02	0.44; 0.49; 0.84; 0.21	Brazil, CL-Brener (Esmeraldo-like), Dm28c, Sylvio, Y
	ASQNVWEDAY	A* 01:01; A* 30:02; B* 15:01	0.25; 0.45; 0.73	Brazil, CL-Brener (Esmeraldo-like), Dm28c, Sylvio
	DENSAYSSVL	B* 18:01; B* 40:01; B* 44:03	0.35; 0.78; 0.96	Brazil, CL-Brener (Esmeraldo-like), Dm28c, Sylvio
	KSVLQSWKNW	A* 32:01; B* 57:01; B* 58:01	0.26; 0.02; 0.05	Brazil, CL-Brener (Esmeraldo-like), Dm28c, Y
	DATPLLGASL	A* 68:02; B* 35:03 B* 51:01	0.86; 0.67; 0.7	-
	MRVMLFTHPL	B* 14:02; B* 27:05 B* 39:01	0.8; 0.98; 0.85	Brazil, CL-Brener (Esmeraldo-like), Dm28c, Sylvio, Y

Supplementary Table 1. Immunodominant epitopes found in TS from TRASP sequence. *In silico* analysis of epitope prediction was used to identify HLA-ABC epitopes present in TS fragment included in TRASP sequence. Also, the conservation of each epitope among different *T. cruzi* strains was evaluated through BLAST.

Supplementary Table 2

	Peptide	HLA-ABC	Percentile Rank (IEDB)	Conservation
ASP-2	YESRDMGTTW	A* 25:01; B* 18:01; B* 38:01; B* 40:01; B* 44:02; B* 44:03; B* 53:01; B* 57:01; B* 58:01	0.39; 0,26; 0.87; 0.81; 0.02; 0.03; 0.36; 0.04; 0.06	Brazil, Dm28c, Sylvio
	KEIESVLRTW	A* 32:01; B* 18:01; B* 40:01; B* 40:02; B* 44:02; B* 44:03; B* 53:01; B* 57:01; B* 58:01	0.21; 0.82; 0.82; 0.73; 0.01; 0.01; 0.88; 0.12; 0.22	-
	RQYSFVNHRF	A* 23:01; A* 24:02; A* 30:02; A* 32:01; B* 15:01; B* 27:05; B* 48:01	0.06; 0.04; 0.46; 0.05; 0.05; 0.7; 0.18	Y
	NETLSNALLY	A* 01:01; A* 26:01; A* 29:02; A* 30:02; B* 18:01; B* 44:02; B* 44:03;	0.18; 0.59; 0.17; 0.57; 0.16; 0.27; 0.2	-
	KNYPFSSITY	A* 23:01; A* 24:02; A* 29:02; A* 30:02; B* 15:01; B* 58:01	0.53; 0.38; 0.21; 0.03; 0.38; 0.95	Sylvio
	TIEGRKVMLY	A* 01:01; A* 26:01; A* 29:02; A* 30:02; B* 44:02; B* 44:03;	0.14; 0.56; 0.49; 0.6; 0.5; 0.59	Brazil, Dm28c, Sylvio, Y
	AISQRGVRSY	A* 01:01; A* 26:01; A* 29:02 A* 30:02; B* 15:01; B* 46:01	0.69; 0.8; 0.97; 0.2; 0.18; 0.54	-
	RTWAQLDAFF	A* 23:01; A* 24:02; A* 32:01; B* 57:01; B* 58:01	0.35; 0.33; 0.21; 0.23; 0.28	Dm28c
	TEWETGQILM	B* 18:01; B* 40:01; B* 40:02; B* 44:02; B* 44:03;	0.15; 0.12; 0.14; 0.33; 0.21	Brazil, CL-Brener (Esmeraldo-like), Dm28c, Sylvio, Y
	FTGPGPMATW	A* 25:01; A* 26:01; B* 53:01; B* 57:01; B* 58:01	0.06; 0.56; 0.3; 0.03; 0.01	-
	TYSTDNGNNW	A* 23:01; A* 24:02; B* 57:01; B* 58:01	0.27; 0.21; 0.47; 0.27	Brazil, Dm28c
	IVMQNNTLVF	A* 23:01; A* 24:02; B* 15:01; B* 46:01	0.93; 0.99; 0.55; 0.34	-
	SNTSSGGNTW	B* 53:01; B* 57:01; B* 58:01	0.81; 0.12; 0.08	Brazil
	LLYSDGNLHL	A* 02:01; A* 23:01; A* 24:02	0.06; 0.51; 0.34	-
	STDNGNNWVF	A* 01:01; B* 57:01; B* 58:01	0.2; 0.87; 0.62	Brazil, Dm28c
	LVFPLVVNGK	A* 03:01; A* 11:01; A* 68:01	0.26; 0.36; 0.42	-
	ATVTKASKVK	A* 03:01; A* 11:01; A* 30:01	0.43; 0.35; 0.83	Brazil
	TPTAGLVGFL	A* 68:02; B* 07:02; B* 35:03	0.48; 0.64; 0.48	Brazil, CL-Brener (Esmeraldo-like), Dm28c, Sylvio, Y

Supplementary Table 2. Immunodominant epitopes found in ASP-2 from TRASP sequence. *In silico* analysis of epitope prediction was used to identify HLA-ABC epitopes present in ASP-2 fragment included in TRASP sequence. Also, the conservation of each epitope among different *T. cruzi* strains was evaluated through BLAST.

Supplementary Table 3

	Peptide	DLA	Percentile Rank (IEDB)
Trans-sialidase	VMLFTHPLNFK	8803401; 8850801	0.28; 0.5
	RIIKSVLQSW	8803401	0.34
	RWLRDRLNLW	8803401	0.58
	RIYNVGQVSI	8850101	0.94

Supplementary Table 3. Epitope prediction of TS sequence for dogs' MHC (DLA). Prediction was performed for the three DLAs available on IEDB: DLA-8803401, DLA-8850101 and DLA-8850801.

Supplementary Table 4

	Peptide	DLA	Percentile Rank (IEDB)
ASP-2	RQYSFVNHRF	8803401; 8850801	0.23; 0.53
	AISQRGVRSY	8803401	0.46
	RTFHVGPVAM	8803401	0.54
	LLYSDGNLHL	8803401; 8850101	0.95; 0.34
	AVNETLSNAL	8850101	0.16
	WVFPESISPV	8850101	0.98
	LVFPLVVNGK	8850801	0.71

Supplementary Table 4. Epitope prediction of ASP-2 sequence for dogs' MHC (DLA). Prediction was performed for the three DLAs available on IEDB: DLA-8803401, DLA-8850101 and DLA-8850801.

Supplementary Table 5

Table 1: Evaluation of Safety and Toxicity in dogs submitted to different immunization protocols

Groups	Time	Weight loss	Fever	Edema	Local pain	Rash	Skin peeling
	24-72h after 1st dose						
PBS		-	-	-	-	-	-
DNAØ + AdβGal		-	-	-	-	-	-
Poly-ICLC		-	-	-	-	-	-
DNA TS/ASP + Ad TS/ASP		-	-	-	-	-	-
TRASP + Poly-ICLC		-	-	-		-	-
	24-72h after 2nd dose						
PBS		-	-	-	-	-	-
DNAØ + AdβGal		-	-	-	-	-	-
Poly-ICLC		-	-	1/6	-	-	-
DNA TS/ASP + Ad TS/ASP		-	-	4/9	-	-	-
TRASP + Poly-ICLC		-	-	6/9	-	-	-
	24-72h after 3rd dose						
PBS		-	-	-	-	-	-
DNAØ + AdβGal		-	-	-	-	-	-
Poly-ICLC[(c)]		-	-	2/6	-	-	-
DNA TS/ASP + Ad TS/ASP		-	-	8/9	1/9	-	-
TRASP + Poly-ICLC		-	-	9/9	2/9	-	-

For each group, the proportion of dogs that presented some of the physiological and/or behavioral changes evaluated, such as: weight loss, fever (rectal temperature above 39.5 °C), changes at the site of inoculation (edema, local pain, rash and skin peeling), for 72 hours after each dose administration.

Supplementary Table 5. Evaluation of safety and toxicity in dogs submitted to different immunization protocols. For each group, the proportion of dogs that presented some of the physiological and/or behavioral changes evaluated, such as: weight loss, fever (rectal temperature above 39.5 °C), changes at the site of inoculation (edema, local pain, rash and skin peeling), for 72 hours after each dose administration.
