# Supplementary material for: ASP-2/Trans-sialidase chimeric protein induces robust protective immunity in experimental models of Chagas’ disease
Source: NPJ Vaccines. 2023 May 31;8:81. doi: 10.1038/s41541-023-00676-0 (PMC10231858; doi:10.1038/s41541-023-00676-0)
Supplement: Supplementary file 2 — Reporting Summary [file 41541_2023_676_MOESM2_ESM.pdf]

nature portfolio

Corresponding author(s): Ricardo T. Gazzinelli (ricardo.gazzinelli@umassmed.edu)

Last updated by author(s): May 3, 2023

Reporting Summary

Nature Portfolio wishes to improve the reproducibility of the work that we publish. This form provides structure for consistency and transparency in reporting. For further information on Nature Portfolio policies, see our Editorial Policies and the Editorial Policy Checklist.

Statistics

For all statistical analyses, confirm that the following items are present in the figure legend, table legend, main text, or Methods section.

n/a	Confirmed	
☐	☒	The exact sample size (*n*) for each experimental group/condition, given as a discrete number and unit of measurement
☐	☒	A statement on whether measurements were taken from distinct samples or whether the same sample was measured repeatedly
☐	☒	The statistical test(s) used AND whether they are one- or two-sided<br>*Only common tests should be described solely by name; describe more complex techniques in the Methods section.*
☐	☒	A description of all covariates tested
☐	☒	A description of any assumptions or corrections, such as tests of normality and adjustment for multiple comparisons
☐	☒	A full description of the statistical parameters including central tendency (e.g. means) or other basic estimates (e.g. regression coefficient) AND variation (e.g. standard deviation) or associated estimates of uncertainty (e.g. confidence intervals)
☐	☒	For null hypothesis testing, the test statistic (e.g. *F*, *t*, *r*) with confidence intervals, effect sizes, degrees of freedom and *P* value noted<br>*Give P values as exact values whenever suitable.*
☒	☐	For Bayesian analysis, information on the choice of priors and Markov chain Monte Carlo settings
☒	☐	For hierarchical and complex designs, identification of the appropriate level for tests and full reporting of outcomes
☒	☐	Estimates of effect sizes (e.g. Cohen's *d*, Pearson's *r*), indicating how they were calculated

Our web collection on statistics for biologists contains articles on many of the points above.

Software and code

Policy information about availability of computer code

Data collection	Flow Cytometry: BD FACSDIVA V8.0.1<br>ELISA:SOFTmaxPRO V4.3.1 LS
Data analysis	Epitope prediction:<br>- The Immune Epitope Database and Analysis Resource (IEDB)<br>- Bimas<br>- SYFPEITHI<br><br>Flow Cytometry: FlowJo V10.5.3<br>Statistics: GraphPad Prism V6.0

For manuscripts utilizing custom algorithms or software that are central to the research but not yet described in published literature, software must be made available to editors and reviewers. We strongly encourage code deposition in a community repository (e.g. GitHub). See the Nature Portfolio guidelines for submitting code & software for further information.

Data

Policy information about availability of data

All manuscripts must include a data availability statement. This statement should provide the following information, where applicable:

- Accession codes, unique identifiers, or web links for publicly available datasets
- A description of any restrictions on data availability
- For clinical datasets or third party data, please ensure that the statement adheres to our policy

The authors declare that all data supporting the findings of this study are available within the paper and its supplementary information files. If any more information is needed, data are available from the corresponding author upon reasonable request. For epitope prediction of Trans-sialidase (accession Q4CZ79) and ASP-2 (accession U77951.1) proteins, we used The Immune Epitope Database (IEDB), Bimas and SYFPEITHI.

Human research participants

Policy information about studies involving human research participants and Sex and Gender in Research.

Reporting on sex and gender	*Use the terms sex (biological attribute) and gender (shaped by social and cultural circumstances) carefully in order to avoid confusing both terms. Indicate if findings apply to only one sex or gender; describe whether sex and gender were considered in study design whether sex and/or gender was determined based on self-reporting or assigned and methods used. Provide in the source data disaggregated sex and gender data where this information has been collected, and consent has been obtained for sharing of individual-level data; provide overall numbers in this Reporting Summary. Please state if this information has not been collected. Report sex- and gender-based analyses where performed, justify reasons for lack of sex- and gender-based analysis.*
Population characteristics	*Describe the covariate-relevant population characteristics of the human research participants (e.g. age, genotypic information, past and current diagnosis and treatment categories). If you filled out the behavioural & social sciences study design questions and have nothing to add here, write "See above."*
Recruitment	*Describe how participants were recruited. Outline any potential self-selection bias or other biases that may be present and how these are likely to impact results.*
Ethics oversight	*Identify the organization(s) that approved the study protocol.*

Note that full information on the approval of the study protocol must also be provided in the manuscript.

Field-specific reporting

Please select the one below that is the best fit for your research. If you are not sure, read the appropriate sections before making your selection.

☒ Life sciences ☐ Behavioural & social sciences ☐ Ecological, evolutionary & environmental sciences

For a reference copy of the document with all sections, see nature.com/documents/nr-reporting-summary-flat.pdf

Life sciences study design

All studies must disclose on these points even when the disclosure is negative.

Sample size	The group sizes for experiments with mice or human samples were determined by power calculations statistical analysis.
Data exclusions	No data were excluded from the analysis.
Replication	The experiments were repeated at least twice, except for dogs experiments, if the statistical significance was P<0.05. All attempts at replication were successful.
Randomization	Animals and samples were randomly allocated.
Blinding	We didn't find the need for blinding analysis because data was objective and phenotypes were very distinct.

Reporting for specific materials, systems and methods

We require information from authors about some types of materials, experimental systems and methods used in many studies. Here, indicate whether each material, system or method listed is relevant to your study. If you are not sure if a list item applies to your research, read the appropriate section before selecting a response.

Materials & experimental systems

n/a	Involved in the study
☐	☒ Antibodies
☒	☐ Eukaryotic cell lines
☒	☐ Palaeontology and archaeology
☐	☒ Animals and other organisms
☒	☐ Clinical data
☒	☐ Dual use research of concern

Methods

n/a	Involved in the study
☒	☐ ChIP-seq
☐	☒ Flow cytometry
☒	☐ MRI-based neuroimaging

Antibodies

Antibodies used

-Goat Anti-Mouse IgG-HRP (SouthernBiotech, Cat 1030-05, Lot B1411-PD01B)
- Goat Anti-Mouse IgG1-HRP (SouthernBiotech, Cat 1070-05, Lot J6908-T229B)
- Goat Anti-Mouse IgG2c-HRP (SouthernBiotech, Cat 1079-05, Lot D6603-X619)
- anti-mouse CD3 PE-Cy5 (145-2C11, BD)
- anti-mouse CD3 APC-Cy7 (145-2C11, BD)
- anti-mouse CD4 Alexa Fluor 700 (RM4-5, eBioscience)
- anti-mouse CD8 Alexa Fluor 700 (53-6.7, BD)
- anti-mouse CD62L APC (MEL-14, BD)
- anti-mouse CD44 BV 605 (IM7, eBioscience)
- anti-mouse CD278/ICOS FITC (7E-1769, BD)
- anti-mouse PD-1 PE-Texas Red (J43, Invitrogen)
- anti-mouse CXCR5 PE-Cy7 (SPRCL5, Invitrogen)
- anti-mouse CXCR3 Pacific Blue (CXCR3-173, BD)
- anti-mouse CD19 FITC (1D3, eBioscience)
- anti-mouse CD27 PE (LG.7F9, eBioscience)
- anti-mouse IgD Pacific Blue (11-26, BD)
- anti-mouse IFN-γ PerCP-Cy5.5 (XMG1.2, eBioscience)
- anti-mouse IFN-γ APC (XMG1.2, eBioscience)
- anti-mouse Bcl-6 Pacific Blue (K112-91, BD)
- anti-canine CD8 Alexa Fluor 647 (YCATE55.9, Bio-Rad)
- anti-canine CD4 FITC (YKIX302.9, Bio-Rad)
- anti-bovine IFN-γ PE (CC302, Bio-Rad)
- goat anti-dog IgG-heavy and light chain - HRP (Cat A40-123P, Bethyl Laboratories)
- goat anti-dog IgG1 - HRP (Cat A40-120P, Bethyl Laboratories)
- sheep anti-dog IgG2 - HRP (Cat A40121P, Bethyl Laboratories)
- goat anti-dog IgM - HRP (Cat BEYA40-116P, Bethyl Laboratories)

Validation

All the antibodies were commercial and validated by the manufacturer. The antibodies for flow cytometry have been tested for mouse splenocytes and were properly titrated before the experiments. The dilutions of antibodies used for ELISA were recommended by the manufacturer and optimized in our experiments.

-Goat Anti-Mouse IgG-HRP (SouthernBiotech, Cat 1030-05, Lot B1411-PD01B)
Specificity: Reacts with the heavy chains of mouse IgG1, IgG2a, IgG2b, IgG2c, and IgG3
Source: Pooled antisera from goats hyperimmunized with mouse IgG
Cross Adsorption: Mouse IgM and IgA; human immunoglobulins and pooled sera; may react with immunoglobulins from other species
Purification: Affinity chromatography on mouse IgG covalently linked to agarose
Applications: ELISA, FLISA, FC
Working dilutions (ELISA): 1:4,000 - 1:8,000
- Goat Anti-Mouse IgG1-HRP (SouthernBiotech, Cat 1070-05, Lot J6908-T229B)
Specificity: Reacts with the heavy chain of mouse IgG1
Source: Pooled antisera from goats hyperimmunized with mouse IgG1
Cross Adsorption: Mouse IgG2a, IgG2b, IgG3, IgM, and IgA; human immunoglobulins and pooled sera; may react with immunoglobulins from other species
Purification: Affinity chromatography on mouse IgG1 covalently linked to agarose
Applications: ELISA, FLISA, FC
Working dilutions (ELISA): 1:4,000 - 1:8,000
- Goat Anti-Mouse IgG2c-HRP (SouthernBiotech, Cat 1079-05, Lot D6603-X619)
Specificity: Reacts with the heavy chain of C57BL/6 mouse IgG2c
Source: Pooled antisera from goats hyperimmunized with mouse IgG2c
Cross Adsorption: Mouse IgG1, IgG2a, IgG2b, IgG3, IgM, and IgA; may react with immunoglobulins from other species
Purification: Affinity chromatography on mouse IgG2c covalently linked to agarose
Applications: ELISA, FLISA
Working dilutions (ELISA): 1:4,000 - 1:8,000

Animals and other research organisms

Policy information about studies involving animals; ARRIVE guidelines recommended for reporting animal research, and Sex and Gender in Research

Laboratory animals	- Female C57BL/6, B2m-/-, mMT, IFN-g-/- mice, 6-10 weeks old<br>- Mongrel dogs (15 males and 21 females)
Wild animals	The study did not involve wild animals.
Reporting on sex	It is well described that male mice are more susceptible to T. cruzi infection (Hauschka, T. S., 1947), therefore our experiments were performed only with female mice.
Field-collected samples	The study did not include samples collected from the field.
Ethics oversight	The protocols for mouse and dog experiments were approved by Fundação Oswaldo Cruz and Universidade Federal de Ouro Preto Ethics Commission on Animal Use (CEUA), LW 02/19 and 2017/37, respectively.

Note that full information on the approval of the study protocol must also be provided in the manuscript.

Flow Cytometry

Plots

Confirm that:

☒ The axis labels state the marker and fluorochrome used (e.g. CD4-FITC).

☒ The axis scales are clearly visible. Include numbers along axes only for bottom left plot of group (a 'group' is an analysis of identical markers).

☒ All plots are contour plots with outliers or pseudocolor plots.

☒ A numerical value for number of cells or percentage (with statistics) is provided.

Methodology

Sample preparation	For imunophenotyping splenocytes derived from immunized mice [51], a total of 2 x 106 cells were incubated for 18h at 37°C and 5% CO2 with RPMI 1640 medium alone or containing10 µg/mL of DTT-1. During the last 6 h of culture, GolgiStop and GolgiPlug Protein Transport Inhibitors (BD Biosciences) were added to the cell cultures. The splonocytes were then washed with PBS, stained with Live/Dead reagent (Invitrogen) and incubated with FcBlock (BD Biosciences). The following mAbs were used to label cell surface markers: anti-CD3 PE-Cy5 or APC-Cy7 (145-2C11, BD), anti-CD4 Alexa Fluor 700 (RM4-5, eBioscience), anti-CD8 Alexa Fluor 700 (53-6.7, BD), anti-CD62L APC (MEL-14, BD), anti-CD44 BV 605 (IM7, eBioscience), anti-CD278/ICOS FITC (7E-1769, BD), anti-PD-1 PE-Texas Red (J43, Invitrogen), anti-CXCR3 Pacific Blue (CXCR3-173, BD), anti-CD19 FITC (1D3, eBioscience), anti-CD27 PE (LG.7F9, eBioscience) and anti-IgD Pacific Blue (11-26, BD). For intracellular staining, cells were washed, fixed and permeabilized according to the manufacturer's instructions (Cytofix/Cytoperm, BD Biosciences or Foxp3/Transcription Factor Staining, eBioscience) and stained with anti-IFN-γ PerCP-Cy5.5 or APC (XMG1.2, eBioscience) and anti-Bcl-6 Pacific Blue (K112-91, BD). Flow cytometry was carried out using a BD LSRFortessa and ~100,000 live CD3+ CD4 + or CD3+ CD8+ cells were acquired. Data were analyzed using FlowJo software.<br>For experiments with dogs, the blood was collected and the incubated with RPMI with PMA (25ng/mL), ionomycin (1µg/mL) and brefeldin A (10 µg/mL, Sigma). For comparison, there were also tubes containing only RPMI and brefeldin A. The cells were then washed and stained with anti-CD8 Alexa Fluor 647 (YCATE55.9, Bio-Rad) and anti-CD4 FITC (YKIX302.9, Bio-Rad). For intracellular staining, cells were permeabilized and stained with anti-IFN-γ PE (CC302, Bio-Rad). The samples were read on the FACSCalibur flow cytometer in which 100,000 events were acquired. The final data is represented by indexes, determined by dividing the percentage of positive cells in the PMA/Ionomycin-stimulated cells divided by the paired unstimulated control.
Instrument	BD LSRFortessa<br>FACSCalibur
Software	Collection of data was performed using the Digital DIVA hardware and software and analysis using FlowJo software
Cell population abundance	No cell sorting data.

Gating strategy

- Splenocytes were gated for singlets (FSC-H X FSC-A), live (SSC-A x Live/Dead), lymphocytes (SSC-A x FSC-A), CD3+ CD8+ (CD3 x CD8), IFN-γ+ (SSC-A x IFN-γ) and with CD44 x CD62L they were separated into effector/effector memory (CD44+ CD62L-) and central memory (CD44+ CD62L+).

- Splenocytes were gated for singlets (FSC-H X FSC-A), live (SSC-A x Live/Dead), lymphocytes (SSC-A x FSC-A), CD3+ CD4+ (CD3 x CD8) and CXCR3+ (SSC-A x CXCR3) and IFN-γ+ (SSC-A x IFN-γ) for CD4+ Th1 or ICOS+ PD-1+ (ICOS X PD-1) and Bcl6+ (SSC-A x Bcl6+) for CD4+ Tfh. For class-switched memory B lymphocytes, cells were gated for singlets (FSC-H X FSC-A), live (SSC-A x Live/Dead), lymphocytes (SSC-A x FSC-A), CD19+ (SSC-A x CD19) and IgD- CD27+ (IgD x CD27).

☒ Tick this box to confirm that a figure exemplifying the gating strategy is provided in the Supplementary Information.
